# Sustained Drug Release from Smart Nanoparticles in Cancer Therapy: A Comprehensive Review

**DOI:** 10.3390/mi13101623

**Published:** 2022-09-28

**Authors:** Xue Bai, Zara L. Smith, Yuheng Wang, Sam Butterworth, Annalisa Tirella

**Affiliations:** 1 Division of Pharmacy and Optometry, School of Health Science, Faculty of Biology, Medicine and Health, University of Manchester, Oxford Road, Manchester M13 9PT, UK; 2 BIOtech-Center for Biomedical Technologies, Department of Industrial Engineering, University of Trento, Via delle Regole 101, 38123 Trento, Italy

**Keywords:** cancer nanomedicine, drug delivery systems, sustained drug release, polymeric nanoparticles, liposomes, microfluidics

## Abstract

Although nanomedicine has been highly investigated for cancer treatment over the past decades, only a few nanomedicines are currently approved and in the market; making this field poorly represented in clinical applications. Key research gaps that require optimization to successfully translate the use of nanomedicines have been identified, but not addressed; among these, the lack of control of the release pattern of therapeutics is the most important. To solve these issues with currently used nanomedicines (e.g., burst release, systemic release), different strategies for the design and manufacturing of nanomedicines allowing for better control over the therapeutic release, are currently being investigated. The inclusion of stimuli-responsive properties and prolonged drug release have been identified as effective approaches to include in nanomedicine, and are discussed in this paper. Recently, smart sustained release nanoparticles have been successfully designed to safely and efficiently deliver therapeutics with different kinetic profiles, making them promising for many drug delivery applications and in specific for cancer treatment. In this review, the state-of-the-art of smart sustained release nanoparticles is discussed, focusing on the design strategies and performances of polymeric nanotechnologies. A complete list of nanomedicines currently tested in clinical trials and approved nanomedicines for cancer treatment is presented, critically discussing advantages and limitations with respect to the newly developed nanotechnologies and manufacturing methods. By the presented discussion and the highlight of nanomedicine design criteria and current limitations, this review paper could be of high interest to identify key features for the design of release-controlled nanomedicine for cancer treatment.

## 1. Introduction

Cancer was responsible for nearly 10 million deaths in 2020 worldwide [1]. Most of the available anti-cancer drug regimens use highly toxic drugs, such as doxorubicin and cyclophosphamide, which are administered to patients systemically. The systemic toxicity of such drugs limits the therapeutic concentrations that are achievable at the target tissue (i.e., cancer) for the vast majority of the compounds in use [2,3]. New delivery modalities have been investigated over the past years aiming to improve the therapeutic index, by enhancing tumour cell targeting while delivering the chemotherapeutic(s). Almost five decades ago, nanomedicines emerged as promising technologies for cancer therapy as they allow for controlled delivery and release of therapeutics. However, after all these years of research, only a few have been approved and are currently used clinically. Over the past two decades, research has focused on the manufacturing of biomaterials in the form of nanocarrier-based drug delivery systems able to improve the therapeutic efficacy of chemotherapeutic agents. Since its discovery, graphene-based 2D materials have also been studied as carriers for the delivery of drugs due to properties such as rapid charge carrier mobility, large surface area and thermal conductivity [4]. This review paper will not discuss graphene-based systems, but will focus on advanced and state-of-the-art three-dimensional nanoparticles (NPs, Table 1) and discuss their modifications to improve efficacy in the context of cancer treatment.

**Figure 1:**
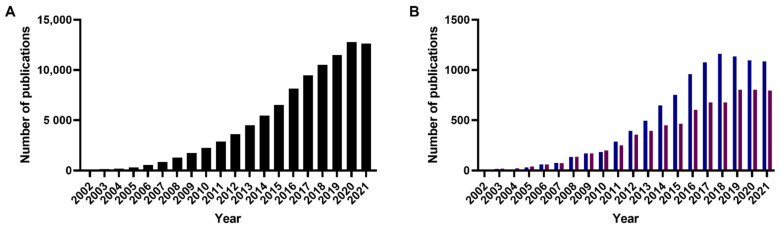
Number of publications published on (**A**) (anti-)cancer nanoparticles since 2002. (**B**) polymeric nanoparticles (blue) and liposomes (red) for cancer treatment since 2002.

A review of research papers published in the past 20 years returned about 87,000 entries published on non-graphene-based nanoparticles for chemotherapeutic delivery by Web of Science (Figure 1). Between them, polymeric nanoparticles were found to be the most studied (10% of publications), followed by liposomes (7.5% of publications) [5]. Despite the high number of nanomedicines reported, the U.S. Food and Drug Administration (FDA), European Medicines Agency (EMA) and other administrations have approved so far around only 20 anti-cancer nanomedicines (Table 2, Table 3, Table 4, Table 5, Table 6, Table 7, Table 8, Table 9 and Table 10), evidencing a limited clinical use of nanotechnologies for cancer treatment [6,7,8].

Despite this large volume of research, an analysis of the approaches used in the past decades evidenced that nanomedicines have typically lacked sufficient drug loading and/or appropriate release kinetics of therapeutic doses at the targeted site. Combined with low cellular uptake, accumulation of nanoparticles in off-target organs (e.g., liver and spleen) and insufficient release of therapeutics could explain the poor translation of this research to clinical oncology [9]. For example, the targetability of conventional nanoparticles relies on passive targeting in which the enhanced permeability and retention (EPR) effect plays an important role. The EPR effect, first described by Matsumura and Maeda in 1986 [10,11], is well described in tumors, wherein the epithelium of the dense vascular network has high permeability, and tumor masses have low lymphatic drainage [10,11], however; EPR effect does not guarantee nanoparticles accumulation, and off-targeting phenomenon may occur [12]. Therefore, the surface modification of nanoparticles could be applied to overcome such limitations by achieving active targeting, which is based on altered gene and protein expression profiles exhibited by malignant cells, such as transferrin overexpress [13]. For this reason, smart drug delivery systems have been recently designed, with the scope to improve: (1) accumulation at the tumour site, by either passive, active even stimuli response mechanisms, and/or (2) control the release of loaded drug using appropriate stimuli [14]. In particular, one strategy used in smart drug delivery systems to further improve the therapeutic efficacy is to control the release of the loaded drug with a known rate over up to several weeks. With this approach, smart drug delivery proposes to solve two main problems associated with conventional nanoparticles: the burst and uncontrolled release of the drug [15]. In addition to the safety and efficacy benefits of controlling drug concentrations within the therapeutic window, this approach will also reduce the dosing frequency and thereby potentially improve patients’ compliance (as only 40–50% of compliance rate on long-term medication therapies is recorded [16]).

This review paper will first discuss in-depth drug release strategies and present the most recently developed smart sustained release nanoparticles tested both in vitro and in vivo. Approved nanomedicines for cancer treatment, as well as nanomedicines currently tested in clinical trials, will be presented with highlights of the design criteria for effective sustained drug release. In particular, attention will be given to polymeric nanotechnologies, discussing future improvements for their translation towards clinical use. Finally, emerging approaches in the design and fabrication of polymeric nanoparticles and smart sustained release nanoparticles to enhance control over drug release will be discussed.

## 2. Smart Sustained Release Nanoparticles

### 2.1. Definition and Advantages of Smart Sustained Release Nanoparticles

**Figure 2:**
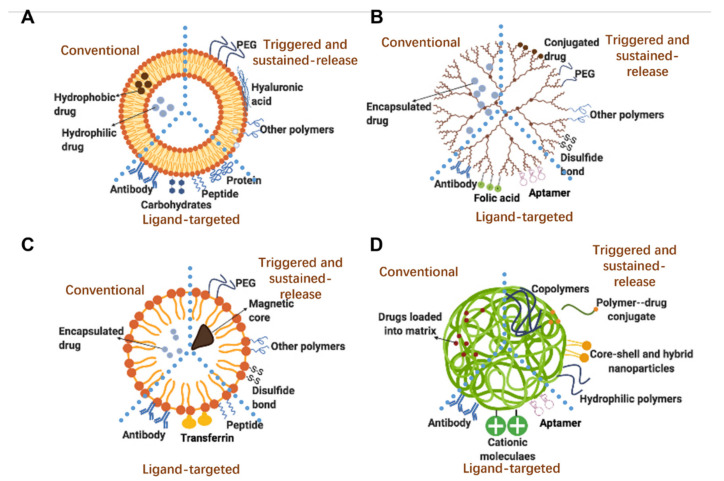
Structures of selected/relevant smart nanoparticles: (**A**) liposomes; (**B**) dendrimers; (**C**) micelles; (**D**) polymeric nanoparticles. Each subfigure highlights the key properties of: conventional, triggered- and sustained-release, and ligand-targeted nanoparticles.

Smart sustained release nanoparticles combine design approaches for the fabrication drug delivery system responsive to external stimuli and allowing for sustained release. Among these, the most represented technologies are: liposomes, dendrimers, micelles and polymeric nanoparticles. The scope is to provide further control over drug release, as summarized in Figure 2 and Table 1.

**Table 1:** Definition and advantages of smart sustained release nanoparticles.

	Smart Drug Delivery System	Sustained Drug Release System
Definition	Release drugs in response to specific physiological triggers, at appropriate time and target site [9].	Deliver drugs at a predetermined rate over an extended period of time [17].
Physiological/clinical benefits	Activation of nanoparticles is controllable which can either accelerate drug release or improve drug retention [18,19]. Overcome biological barriers [18]. Enhance specific targeting and internalization, reduce side effect [18,19]. Deliver multiple therapeutic agents for combination therapy, circumvent multidrug resistance [20]. Improve physiochemical and physiological properties of drugs, such as expanding therapeutic window of drugs and achieving longer half-life over conventional drugs [21,22].	Reduce concentration fluctuation in steady-state drug levels, minimize “peak and valley” pattern [23]. Avoid initial “burst release”, which results in negative therapeutic effects [15]. Decrease drug administration and treatment period, improve patients’ compliance. Maximum utilization of drug, increase safety margin of drug and decrease therapeutic costs [23]. Better life-cycle management of drugs, extended relief of symptoms [9].

### 2.2. Latest Formulation Strategies in the Smart Sustained Release Nanoparticles for Clinical Cancer Treatment

#### 2.2.1. Smart Sustained Release Liposomes

Liposomes, discovered by Alec D. Bangham in 1965 [24], are spherical carriers composed of phospholipids and cholesterol that form a lipid bilayer surrounding an aqueous core (Figure 2A). In contrast to other nanoparticles, liposomes have the unique ability to encapsulate both hydrophilic and hydrophobic compounds, respectively encapsulated in the aqueous and the lipid bilayer [24]. As the lipid bilayer possesses a structure similar to the mammalian cell membrane, enhancement of liposome’s cellular uptake compared to other nanoparticles has been hypothesized [24]. Figure 2A summarizes the evolution of liposomes lipid bilayer characteristics, from the early conventional ‘plain’ phospholipid liposomes, through the inclusion of poly (ethylene glycol) (PEG) [24] to prolong circulating time, and finally to the more recent antibody-targeted immunoliposomes, which target cancer cells to increase treatment efficacy [25].

**Figure 3:**
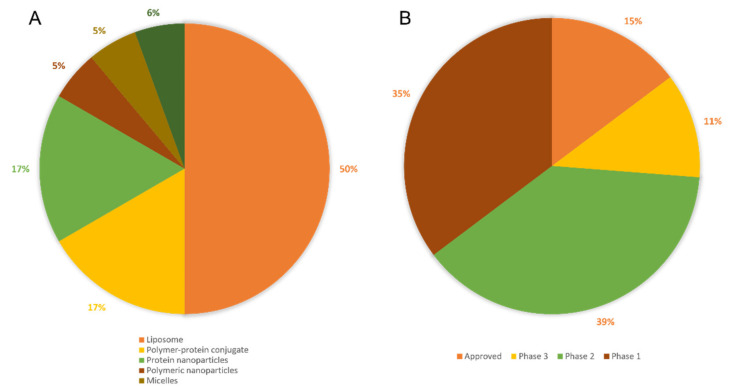
Distribution of (**A**) Nanoparticles approved in clinical use; (**B**) Current statues of all types of clinically used nanoparticles.

In addition to targetability, advancements in the formulation of liposomes have shown the capability to release the therapeutic with prolonged profiles (up to several weeks) [26] and upon stimuli from an external energy source (e.g., thermal) [27,28]. As an example of smart sustained release liposomes, Sun and co-workers synthesized hyaluronic acid-coated, peptide-modified liposomes loading curcumin and celecoxib. Compared to the unmodified liposomes, this formulation is able to prolong the drug release up to 72 h with a recorded release of 77.4% celecoxib and 73.5% curcumin in the presence of hyaluronidase [29]. Of note, liposomes are the most clinically used nanoparticles (Figure 3A), with 9 approved liposomes used for cancer treatment (Table 2). Recently, the first thermo-sensitive smart sustained-release liposomes were reported to be successfully used in clinical trials to treat cancer with enhanced local drug delivery [30,31].

#### 2.2.2. Smart Sustained Release Dendrimers

Dendrimers are 3D branched molecules composed of a dense core and branching layers with functional groups as the outer shell. Dendrimers have been used for the delivery of several molecules of interest, and advanced modifications of polymers are used to improve the formulation of different dendrimers and delivery profiles (Figure 2B) [32].

As with many other nanoparticles, the release of loaded molecules occurs within the first 24 h. Recently, smart sustained release dendrimers (i.e., redox and pH-responsive dendrimer-heparin conjugates loaded with letrozole) were described to release payloads over 100 h, with release rate controlled by pH and concentration of glutathione. Letrozole was released with a higher rate when incubated at higher concentrations of glutathione, and at lower pH (i.e., 4.5) due to increased cleavage of disulphide linkage in dendrimers [33]. Recent success on the use of dendrimers clinically was reported for DEP^®^ docetaxel, a formulation of PEGylated poly(L-lysine) dendrimer containing docetaxel conjugated to its surface, reported to have progressed to phase I clinical trials for the treatment of solid tumours [34].

#### 2.2.3. Smart Sustained Release Micelles

Micelles are colloidal suspensions formed by the aggregation of amphiphilic molecules, or surfactants [14]. Amphiphilic molecules are composed of a hydrophilic and a hydrophobic domain, displaying unique self-assembly characteristics when dissolved in different solvents (Figure 2C). In water-based solutions and at a critical concentration of surfactants, the hydrophobic domain of surfactant assembles and begins to form cores; whereas the hydrophilic domain interacts with the solvent and stabilizes the micelle. In the case of hydrophobic solvent, this effect is reversed, as micelles with hydrophilic cores and hydrophobic shells self-assemble with the increase of surfactant concentration and when the critical micelle concentration is reached. As a function of the composition of the amphiphilic molecule, the region in contact with the solvent can present different characteristics, which can be useful for the design of smart sustained release nanoparticles, such as pH gradient-dependent release [35]. For drug delivery purposes micelles are typically formulated with a hydrophobic core encapsulating hydrophobic drugs, with functionalities such as folic acid, glucose and monoclonal antibodies decorating the hydrophilic region [14]. Yu et al. [35] reported on the synthesis of pH-sensitive smart sustained-release micelles to release doxorubicin. A four-arm star polymer, poly(e-caprolactone)-b-poly(2-(diethylamino)ethylmethacrylate), was used to form micelles. Drug release studies performed at different pH values, showed the release of 24.5% of loaded doxorubicin after 108 h at pH 7.4 (normal physiological condition). Increased release of doxorubicin was observed up to 51.8%, when the pH was reduced to mimic the value of the tumour environment (i.e., pH 5.0) after the same period of time. Micelles currently used clinically and tested in clinical trials as anti-cancer therapies are reported in Table 4.

#### 2.2.4. Smart Sustained Release Polymeric Nanoparticles

The name polymeric nanoparticle typically refers to nanospheres and nanocapsules composed of a polymeric matrix. Nanospheres are solid particles in which molecules are adsorbed on the surface or encapsulated within the polymeric matrix (Figure 2D). Nanocapsules are instead vesicular systems in which therapeutic agents are encapsulated inside the core; in these, a polymeric membrane (shell) protects the payload within the core from environmental factors [36]. Typically polymeric nanoparticles release drugs within a short period of time; however, recent studies showed the use of polymeric nanoparticles for controlled and sustained drug release [37,38,39]. Gao et al. [39] described the use of erythrocyte membrane-wrapped pH-sensitive polymeric nanoparticles for the released of paclitaxel. Higher concentrations of paclitaxel can be released at pH 6.5 with a sustained pattern (~30% release at 108 h) when compared to the release at pH 7.4. Moreover, thanks to the erythrocyte membrane coating, these nanoparticles increased substantially the intravenous circulation time with lower immunogenicity compared to uncoated nanoparticles [39]. Throughout this review paper, nanoparticles made of poly (d, l-lactide-*co*-glycolide) (PLGA) and poly-(lactic acid) (PLA) will be thoroughly discussed, as these are FDA-approved polymers which are known to be highly biocompatible and biodegradable, with low reported toxicity. Moreover, PLGA, PLA and their blends have been widely used as polymeric matrices in many drug delivery applications [40].

**Table 2:** Liposomes approved for clinical use. PEG = poly (ethylene glycol); AIDS = acquired immunodeficiency syndrome; FDA = U.S. Food and Drug Administration; EMA = European Medicines Agency; HIV = human immunodeficiency virus; DSPC = distearoyl-phosphatidylcholine; EPR = enhanced permeability and retention; DOPS = dioleoylphosphatidylserine; DOPC = dioleoyl-phosphatidylcholine.

Product Name	Formulation	Drug/Therapeutic Agent	Treatment	Status	Formulation Properties	Ref.
Doxil	PEGylated liposomal	Doxorubicin	Various cancer types	Approved by FDA (1995)	Passive-targeting formulation. Sustained drug release achieved by prolonged dissolution rate of drug crystals in the core of the liposome.	[41,42]
DaunoXome	Liposomal	Daunorubicin	HIV-associated Kaposi’s sarcoma	Approved by FDA (1996)	Daunorubicin in small unilamellar vesicles composed of DSPC and cholesterol in a 2:1 mole ratio with 45 nm average size. Drug release over a prolonged period (36 h or more).	[43,44,45]
Myocet	Liposomal	Doxorubicin	Metastatic breast cancer	Approved by EMA (2000)	Passive-targeting formulation. Loading technique involves a pH gradient and citrate complex leading to the high ratio of drug to lipid.	[46,47]
Lipo-dox	PEGylated liposomal	Doxorubicin	Metastatic breast cancer, ovarian cancer and AIDS-related Kaposi’s sarcoma	Approved by Department of Health of Taiwan (2002)	Lipid composition includes DSPC to reduce drug leakage during preparation and enhance liposomes stability.	[46]
Lipusu	Liposomal	Paclitaxel	Breast and non-small-cell lung cancer	Approved in China (2003)	Lipusu instead of conventional paclitaxel has been shown to have a markedly reduced toxicity while retaining equal efficacy in cancer models	[48]
CPX-351	Liposomal	Cytarabine and daunorubicin (5:1 molar ratio)	Acute myeloid leukemia	Approved by FDA (2007)	Gel state at body temperature, providing stability and controlled drug release with limited systemic drug distribution.	[49]
Mepact	Liposomal	Mifamurtide	Osteosarcoma	Approved by EMA (2009)	Size < 100 nm with DOPS: POPC = 3:7 molar ratio, designed to target macrophages (phosphatidyl serine containing lipids provides signal to macrophages). The drug shows no cytotoxicity to normal or tumour cells in vitro.	[50]
Marqibo	Liposomal	Vincristine	Leukemia	Approved by FDA (2012)	SM and cholesterol as the liposomal carrier with the size around 100 nm. Increased extravasation into tumours and sustained drug release (approximately 18–39% release of encapsulated drug at 24 h at 37 °C).	[51,52]
Onivyde	Liposomal	Irinotecan	Metastatic pancreatic cancer	Approved by FDA (2015)	Sustained-release formulation could target tumour by EPR effect. Increased in vivo stability of drug, extended the circulation time.	[53]

**Table 3:** Liposomes currently tested in clinical trials. PEG = poly (ethylene glycol); mPEG = methoxypolyethylene glycol; PK = pharmacokinetics; DOPC = dioleoyl-phosphatidylcholine; HSPC = hydrogenated soy phosphatidylcholine; TfR = transferrin receptor; scFV = single-chain antibody fragments; NGPE = *N*-glutarylphosphatidylethanolamine; EGFR = epidermal growth factor receptor; RNAi = RNA interference; siRNA = small interfering RNA; AON = antisense oligodeoxynuleotides; EE% = encapsulation efficiency; DC = dendritic cells; HER2 = human epidermal growth factor receptor 2; BCL-2 = B-cell lymphoma 2; EPHA2 = type A ephrin receptor 2; NSCLC = non-small-cell lung cancer; APCs = antigen-presenting cells; HLA = human leukocyte antigen; ATRA = all-trans retinoic acid; DMPC = 1,2-dimyristoylphosphatidylcholine; DMPG = 1,2-dimyristoylphosphatidylglycerol; ESM = egg sphingomyelin; Grb-2 = growth factor receptor bound protein 2; MTD = maximum tolerated dose; sPLA2 = secretory phospholipase A2; DSPC = distearoyl-phosphatidylcholine; DSPG = distearoyl phosphatidylglycerol; DSPE = distearoyl phosphatidylethanolamine; miRNA = microRNA; DOTIM = (1-[2-(oleoyloxy)ethyl]-2-oleyl-3-(2-hydroxyethyl) imidazolinium chloride).

Product Name	Formulation	Drug/Therapeutic Agent	Treatment	Status	Formulation Properties	Ref.
ThermoDox	Heat-sensitive liposomal	Doxorubicin	Hepatocellular carcinoma	Phase III	Thermo-sensitive lipids to high temperatures. Drug release is controlled by mild increases in temperature (39.5–43 °C).	[30,31]
S-CKD602	PEGylated liposomal	CKD602	Various cancer types	Phase II	Stealth liposome formulation, composed of phospholipids covalently linked to mPEG, leading to prolonged plasma exposure and superior tumour delivery.	[54,55]
CPX-1	Liposomal	Irinotecan and floxuridine	Colorectal cancer	Phase II	Irinotecan and floxuridine in a fixed 1:1 molar ratio. CPX-1 overcomes the different PK of a single drug and can continue to maintain this ratio after intravenous injection.	[56,57,58]
LE-SN38	Liposomal	SN-38	Metastatic colorectal cancer	Phase II	Improved therapeutic index, efficacy and safety of insoluble SN-38. 50:40:10 molar ratio of DOPC, cholesterol and cardiolipin and a drug to lipid ratio of 1:18. Provide active drugs without conversion by using NeoLipid^®^ patented technology.	[54,59,60]
INGN-401	Liposomal	FUS1	Lung cancer	Phase I	Targeted gene delivery of FUS1 tumour suppressor protein by “plasmid gene expression cassette”, which contains DNA encoding the FUS1 protein. The tightly wrap by cholesterol provides protection against the body’s defense mechanisms.	[61,62]
SPI-077	PEGylated liposomal	Cisplatin	Head and neck cancer, lung cancer, ovarian cancer	Phase II	Long-circulating and sterically stabilized liposomes. Composed of neutral lipids with 110 nm size, and cisplatin to total lipid ratio is 1:70.	[63,64,65]
OSI-7904L	Liposomal	Thymidylate synthase inhibitor	Various cancer	Phase II	Manufactured by HSPC and cholesterol with OSI-7904 loaded in the aqueous cores, size 20–80 nm. Improved the efficacy and increased the half-life.	[64,66,67]
OSI-211	Liposomal	Lurtotecan	Lung cancer, recurrent ovarian cancer	Phase II	Encapsulation of lurtetecan, an inhibitor of the mammalian topoisomerase I enzyme. Increased plasma residence time, improved biodistribution and therapeutic index of the drug.	[64,68,69,70]
SGT-53	TfR-targeting liposomal	Wild-type p53 plasmid DNA	Solid tumours, glioblastoma, metastatic pancreatic cancer	Phase II	Active targeting formulation decorated with anti-TfR scFv as tumour targeting domain. Cationic liposomes internalized by receptor-mediated endocytosis.	[56,71,72]
MBP-426	TfR-targeting Liposomal	Oxaliplatin	Gastric, oesophageal and gastro-oesophageal adenocarcinoma	Phase I/II	Active targeting formulation decorated with human transferrin ligand. pH-responsive liposomes due to the NGPE coating. The layer ensures rapidly disintegration of the particles under acidic conditions.	[56,73,74]
Anti-EGFR-IL-DOX	EGFR-targeting liposomal	Doxorubicin	Breast cancer	Phase II	Active targeting formulation decorated with Fab’ fragment of the anti-EGFR-antibody C225 to target EGFR expressing cells.	[56,75,76]
Atu027	Liposomal	siRNA against protein kinase N3	Advanced or metastatic pancreatic cancer	Phase I/II	Liposomes for RNAi therapy, delivering siRNA to silence the expression of protein kinase N3 in vascular endothelium.	[56,77,78]
DC-Chol-EGFR	Liposomal	EGFR antisense	Head and Neck cancer	Phase I	Cationic liposomes loading EGFR antisense sequence.	[79,80]
EndoTAG-1	Liposomal	Paclitaxel	Pancreatic cancer, liver metastases and HER2-negative and triple-negative breast cancer	Phase III	Cationic liposomes to target angiogenic endothelial cells in solid tumours.	[56,69,81,82,83]
LErafAON	Liposomal	c-Raf ANO	Advanced solid tumour, advanced malignancy	Phase I	Cationic liposomes loading negatively charged c-raf-1 AON with the EE% > 85%. Average size of 400 nm. Pre-clinical analysis of LErafAON showed Raf-1 inhibition and tumour regression.	[80,84,85,86]
Lipoplatin	PEGylated liposomal	Cisplatin	Pancreatic cancer	Phase III	High EE% (95–97%), observed induction of tumor cell apoptosis with 200-fold higher concentration of cisplatin in tumours than free drug. Induced apoptosis to the endothelium of tumor vasculature, hence, portraying strong antiangiogenesis properties.	[54,69,87]
Lipovaxin-MM	DC-targeted liposomal	Melanoma antigens	Malignant melanoma	Phase I	Active targeting liposomes decorated with a multicomponent and multivalent DC targeting allogeneic melanoma.	[56,88,89]
MM-302	HER2-targeted PEGylated liposomal	Doxorubicin	HER2-positive breast cancer	Phase II/III	Active targeting liposomes decorated with 45 single-chain anti-HER2 antibodies (scFv) targeting HER2-overexpressing tumour cells.	[56,90]
PNT2258	Liposomal	DNA oligonucleotide against BCL-2	Relapsed or refractory non-Hodgkin lymphoma and diffuse large B-cell lymphoma	Phase II	pH-responsive formulation, anionic at physiological pH. Average size of 130 nm.	[56,91,92]
Promitil	PEGylated liposomal	Mitomycin C	Advanced solid tumours	Phase I	Significantly lower toxicity profile in preclinical and phase 1 clinical investigations. Drug release is based on the cleavable dithiobenzyl bridge between Mitomycin C and glycerol lipids by reducing agents in tumours.	[93,94,95]
siRNA-EPHA2-DOPC	Liposomal	siRNA against EPHA2	Advanced solid cancers	Phase I	Neutral liposomes loading siRNA to silence EPHA2 and to inhibit tumour cells growth.	[56,96,97]
Tecemotide	Liposomal	Mucin 1 antigen	NSCLC	Phase III	Lipopeptide, encapsulated with MPL and three different lipids in multilayer liposomes, designed to promote APCs uptake so that the peptide is processed via class I and class II HLA moleculesin and triggering cytotoxic T-lymphocytes-mediated mucin 1-specific cellular immune responses.	[56,98,99]
Aroplatin	Liposomal	Cisplatin analog	Various cancers	Phase II	NDDP loaded multi-layer liposomes, synthesized by mixing DMPC and DMPG lipids with acidified salt solution. Note: this is the first liposomal formulation entered into clinical study for delivery of cisplatin analogs.	[54,100,101]
LEP-ETU	Liposomal	Paclitaxel	Ovarian, breast and lung cancers	Phase II	150 nm in size. Liposome carriers have 90:5:5 molar ratio of DOPC, cholesterol and cardiolipin. Drug to lipid molar ratio is 1:33. Maximum drug EE% is 85%.	[54,64,102,103]
Atragen	Liposomal	Tretinoin	Acute promyelocytic leukemia	Phase II	Liposomes composed of retinoic acid, DMPC and soybean oil, containing tretinoin as 2 mg/mL. Compared to free ATRA, this formulation can avoid liver microsomal clearance and show lower in vivo systemic toxicity.	[54,64,104]
Liposomal annamycin	Liposomal	Annamycin	Acute lymphocytic leukemia	Phase I/II	7:3 molar ratio of DMPC:DMPG as the carriers loaded with Anamycin which could intercalate DNA and inhibit topoisomerase II, thereby inhibiting DNA replication and protein synthesis.	[54,105,106]
INX-0076	Liposomal	Topotecan	Advanced solid tumours	Phase I	45:55 molar ratio of cholesterol and ESM. INX-0076 is developed by sphingosomal platform, a novel platform for improved tumour targetability and the duration of exposure of loaded anticancer agents.	[54,107]
INX-0125	Liposomal	Vinorelbine tartrate	Advanced solid tumours	Phase I	45:55 molar ratio of cholesterol and ESM. Based on the sphingosomal platform as INX-0076.	[54]
LEM-ETU	Liposomal	Mitoxantrone	Various cancers	Phase I	90:5:5 molar ratio of DOPC, cholesterol and cardiolipin. Cardiolipin, a negatively charged diphosphatidyl glycerol lipid, forms electrostatic interactions with the loaded drug leading to higher drug loading when compared to other liposome formulations.	[54]
Liposomal Grb-2	Liposomal	Grb-2	Various cancers	Phase I	Neutrally-charged DOPC formulation loading with an antisense oligonucleotide which is designed to inhibit the production of Grb-2.	[54,108,109,110]
Lipoxal	Liposomal	Oxaliplatin	Advanced gastrointestinal cancer	Phase I/II	Lipoxal had a half-life of 24–35 h in humans and MTD of 300 mg/m^2^. Reduced adverse reactions without reducing effectiveness, compared to oxaliplatin.	[111,112,113]
LiPlaCis	PEGylated liposomal	Cisplatin	Solid tumours	Phase I/II	The first controlled-release liposomal formulation encapsulated with cisplatin and modified with sPLA2, a tumour selective enzyme. LiPlaCis liposomes composed of DSPC/DSPG/DSPE-PEG2000 lipids.	[101,114,115,116]
DPX-0907	Liposomal	Multi-tumour associated antigens	HLA-A2-positive advanced stage ovarian, breast and prostate cancer	Phase I	DPX-0907 contains a polynucleotide-based adjuvant, a universal T helper peptide and seven tumour-specific HLA-A2-restricted epitopes could show efficient induction of immune response to cancer peptides.	[56,117,118]
dHER2 + AS15	Liposomal	Recombinant HER2, dHER2, antigen and AS15 adjuvant	Metastatic breast cancer	Phase I/II	Liposomal formulation containing three immune stimulating ingredients: dHER2 is a truncated form of the HER2 protein; AS15 is an immune adjuvant.	[56,119]
MRX34	Liposomal	miRNA-34a mimics	Primary liver cancer, solid tumours and haematological malignancies	Phase I	Composed of amphoteric lipids which confer positive charges to ensure an effective encapsulation of negatively charged miRNA-34a mimics. Liposomes have size of 110 nm and are anionic at neutral pH to minimize particle aggregation and electrostatic adhesion to the cell membrane of endothelial cells.	[56,120]
JVRS-100	Liposomal	Plasmid DNA	Relapsed or refractory leukaemia	Phase I	Liposomes containing cationic lipid DOTIM and neutral lipid cholesterol on the membrane. JVRS-100 stimulate innate immune response to the presence of unmethylated CpG motif in the loaded plasmid.	[121,122]

**Table 4:** Micelles approved for clinical use and currently tested in clinical trials. PEG = poly (ethylene glycol); PLA = poly (lactic acid); MTD = maximum tolerated dose; EMA = European Medicines Agency; PASA = polyaspartic acid; PGlu = polyglutamic acid; DACH-Pt = 1,2-diaminocyclohexane platinum; PAH = phenylalanine hydroxylase.

Product Name	Formulation	Drug/Therapeutic Agent	Treatment	Status	Formulation Properties	Ref.
Genexol-PM	Polymeric micelle	Paclitaxel	Breast cancer and small cell lung cancer	Approved in Korea (2007)	PEG-PLA block copolymers, with size of 20–50 nm. MTD 3 times higher when compared to paclitaxel.	[123]
Apealea	Polymeric micelles	Paclitaxel	Ovarian cancer	Approved by EMA (2018)	Cremophor^®^-free micellar formulation based on the patented excipient platform XR-17. Size 20–60 nm. Excipient ratio 1.3:1.	[124]
NC-6004	Polymeric micelle	Cisplatin	Various cancers	Phase II/III	Mean diameter of around 30 nm and about 39 wt% drug loading. The free platinum is released in the presence of chloride ions. In 0.9% NaCl solution, only 19.6% and 47.8% platinum release at 24 h and 96 h at 37 °C, respectively.	[125]
NK-105	Polymeric micelle	Paclitaxel	Metastatic or recurrent breast cancer	Phase III	A “core-shell-type” polymeric micelles made by block copolymers consisting of PEG and PASA. Size around 85 nm and 23 wt% drug loading.	[126]
NK-911	Polymeric micelles	Doxorubicin	Metastatic pancreatic cancer	Phase II	Doxorubicin-conjugated PASA/PEG nanocarrier with size of 40 nm. Note: NK-911 is the first micellar formulation tested in humans.	[46]
NK-012	Polymeric micelles	SN-38	Advanced solid tumour	Phase II	PEG-PGlu (SN-38) amphiphilic block copolymer. SN-38 covalently linked to PGlu segment with average size of 20 nm.	[127,128]
SP1049C	Polymeric micelles	Doxorubicin	Advanced gastric cancer	Phase III	Pluronic L61 and Pluronic F127 block copolymers with doxorubicin physically loaded. Size around 22–27 nm. Pre-clinical analysis showed SP1049C therapy effectively suppresses the tumorigenicity and aggressiveness	[127,129,130]
NC-4016	Polymeric micelles	Oxaluplatin	Advanced solid tumours	Phase I	Polymer-metal complexes of DACH-Pt and PEG-PGlu block copolymers. NC-4016 has around 15 h blood circulation half-life, with size around 40 nm and 32 wt% drug loading.	[127,131,132,133]
Lipotecan	Polymeric micelles	TLC388 (Camptothecin analog)	Various cancer	Phase I/II	TLC388 has a unique lactone ring modification. Other formulation properties not found.	[134,135]
NC-6300	PEG-b-PAH polymeric micelles	Epirubicin	Solid tumours and soft tissue sarcoma	Phase I/II	PEG-polyaspartate block copolymer linked to Epirubicin by an acid-labile hydrazone bond, particle size of 60–70 nm. The block copolymers are partially substituted by hydrophobic benzyl groups to stabilize the micellar structure.	[136,137]

**Table 5:** Polymeric nanoparticles approved for clinical use and currently tested in clinical trials. PNPs = polymeric nanoparticles; FDA = U.S. Food and Drug Administration; PLGA = poly (d, l-lactide-*co*-glycolide); PSMA = prostate specific membrane antigen; NSCLC = non-small-cell lung cancer; mCRPC = metastatic castration-resistant prostate cancer; PEG = poly (ethylene glycol); PLA = poly (Lactic acid); PEBCA = polyethylbutylcyanoacrylate; PBCA = polybutylcyanoacrylate; mPEG = methoxypolyethylene glycol; siRNA = small interfering RNA; eIF5A = Eukaryotic translation initiation factor 5A; PEI = polyethylenimine.

Product Name	Formulation	Drug/Therapeutic Agent	Treatment	Status	Formulation Properties	Ref.
Eligard	PLGA PNPs	Leuprolide acetate	Prostate cancer	Approved by US FDA (2002)	Leuprolide acetate is administered via an implanted depot delivery system, which releases the drug in a controlled manner over defined intervals—1, 3, 4, or 6 months. Drug loading 4–6%.	[138,139,140]
BIND-014	PSMA-targeting PEG-PLA PNPs	Docetaxel	NSCLC and mCRPC	Phase II	PEG-PLA copolymer nanoparticles physically loaded with docetaxel (drug loading around 10%) with a targeting small-molecule ligand specific for PSMA. Size around 100 nm. Note: BIND-014 is the first-in-man targeted and controlled-release nanoparticles for cancer therapy.	[141,142]
Transdrug	PEBCA PNPs	Doxorubicin	Hepatocellular carcinoma	Phase III	A molecular complex of doxorubicin adsorbed on PEBCA with size of 100–200 nm. 12-fold increase in drug exposure within the hepatic tumor tissue as compared to free doxorubicin.	[106,143,144]
DHAD-PBCA-NPs	PBCA PNPs	Mitoxantrone	Hepatocellular carcinoma	Phase II	Nanoparticles synthesized by PBCA, a biodegradable and bioavailable polymer, with a size of 55 nm and drug loading of 46.77%.	[145,146]
Docetaxel-PNP	PNPs	Docetaxel	Solid tumours	Phase I	PLA-COONa, and copolymer mPEG-PLA nanoparticles physically loaded with docetaxel. Other formulation properties not found.	[147,148]
SNS01-T	PNPs	siRNA against eIF5A and plasmid expressing eIF5A-K50R	Relapsed or refractory B cell malignancies	Phase I/II	Rod-shaped PEI nanoparticles loaded with both siRNA targeting eIF5A1 and an overexpression plasmid expressing the non-modifiable eIF5A-K50R mutant under the regulation of B-cell specific promoter. Average size of 72 nm.	[56,149,150]

**Table 6:** Lipid nanoparticles currently tested in clinical trials. siRNA = small interfering RNA; VEGF = vascular endothelial growth factor; KSP = kinesin spindle protein; DsiRNA = Dicer substrate siRNA; shRNA = short hairpin RNAs; STMN1 = stathmin 1; SNALP = stable nucleic acid lipid particle; RNAi = RNA interference; PLK1 = polo-like kinase 1.

Product Name	Formulation	Drug/Therapeutic Agent	Treatment	Status	Formulation Properties	Ref.
ALN-VSP	Lipid nanoparticle	siRNA	Liver cancer	Phase I	Combination of VEGF siRNA and KSP siRNA in a ratio of 1:1. Size around 80 nm with the neutral charged at physiologic pH. Note: the first lipid nanoparticle-formulated siRNA therapeutic to be tested in cancer patients.	[151,152]
DCR-MYC	Lipid nanoparticle	siRNA against MYC	Hepatocellular carcinoma	Phase I/II	DsiRNA encapsulated within an EnCoreTM lipid nanoparticle targeting c-Myc overexpressed cancerous cells. Note: the first siRNA therapeutic regimen targeting c-Myc that was evaluated clinically.	[153]
pbi-shRNA STMN1 LP	Lipid nanoparticle	shRNA against STMN1	Advanced and/or metastatic cancer	Phase I	Cationic lipid particle loaded with a proprietary RNAi construct consisting of bifunctional shRNA against human STMN1. Other formulation properties not found.	[56,154,155]
TKM-080301	Lipid nanoparticle	Anti-PLK1 siRNA	Various cancers	Phase I/II	SNALP loading siRNA targeting PLK1. Other properties not found.	[150,156,157]

**Table 7:** Polymer-drug conjugate currently tested in clinical trials. PGA = polyglutamic acid; HPMA = *N*-(2-Hydroxypropyl)methacrylamide; NSCLC = non-small cell lung cancer; Mw = molecular weight; DACH = diaminocyclohexane; MAG = methacrylglycinamide; GFLG = glycine-phenylalanine-leucine-glycine; AMA = amidomalonic acid; CMD = carboxymethyldextran; CM = carboxymethyl; DHA = docosahexaenoic acid. AUC = area under the curve.

Product Name	Formulation	Drug/Therapeutic Agent	Treatment	Status	Formulation Properties	Ref.
Xyotax	PGA-Paclitaxel conjugate	Paclitaxel	Ovarian cancer	Phase III	PGA-Paclitaxel conjugation via an ester bond. Xyotax is highly water-soluble, with 37% drug loading. Paclitaxel is released by hydrolysis up to 14% in 24 h in physiological conditions, release is accelerated by lysosomal cathepsin B after endosomal uptake.	[74,158]
Prolindac	HPMA-DACH-platinum conjugate	DACH-platinum	Solid tumours	Phase II	DACH-platinum moiety conjugated with HPMA polymer via a pH-sensitive linker. Compared to unconjugated platinum drugs, AP5346 has a longer half-life and could release drug in acidic condition. Drug loading around 10 wt%.	[159,160]
EP0057	PEG-Cyclodextrin-camptothecin conjugate	Camptothecin	Various tumours	Phase I/II	Cyclodextrin–PEG copolymer chemically conjugated to camptothecin. Drug loading around 10 wt%, and size of 20–60 nm. PEGylation increased residence time in the bloodstream and increased anti-tumour activity.	[74,161,162]
CRLX301	PEG-Cyclodextrin-doxetaxel conjugate	Doxetaxel	Advanced solid tumours	Phase I/II	Cyclodextrin–PEG copolymer chemically conjugated to doxetaxel. Average size of 10–30 nm. Enhanced efficacy and improved pharmacokinetics, longer half-life and more than 20-fold higher drug concentration in tumour tissue, compared to doxetaxel.	[93,163,164]
PK1 (FCE28068)	HPMA-doxorubicin conjugate	Doxorubicin	Breast cancer, NSCLC, colorectal cancer	Phase III	HPMA copolymer covalently linked to doxorubicin via a peptidyl linker. Link is designed to be cleaved by lysosomal enzymes, with drug release after internalization. Polymer Mw 30 kDa. Total doxorubicin 6–8 wt%; free doxorubicin < 1% in respect of total.	[46,165]
PK2 (FCE28069)	HPMA-doxorubicin conjugate	Doxorubicin	Primary or metastatic liver cancer	Phase II	HPMA polymer conjugated to galactose residues and doxorubicin. Synthesized by a 27 kDa HPMA copolymer derivatized with 6.5% mol/wt, <2% free doxorubicin, and 2% mol/wt galactose. Note: PK2 has a similar structure to PK1, with the inclusion of galactosamine to specifically target hepatic cells.	[46,166]
PNU166945	Polymer-drug conjugate	Paclitaxel	Solid tumours	Phase I	An HPMA copolymer-paclitaxel conjugate with the similar structure as PK1, but paclitaxel is conjugated to the terminal glycine by an ester bond.	[166]
MAG-CPT	MAG-camptothecin conjugate	Camptothecin	Various cancers	Phase I	MAG-campothecin conjugated via water soluble link. Average Mw of 18 kDa, and 10 wt% loading of camptothecin.	[135,167,168]
AP5280	HPMA copolymer-platinum conjugate	Carboplatin platinate	Various cancers	Phase I/II	Platinum is linked to a HPMA backbone via a tetrapeptide spacer GFLG and an AMA chelating agent. Drug loading around 8.5 wt%.	[158,169,170]
CT-2106	PGA-camptothecin conjugated	Camptothecin	Solid tumour, malignancies	Phase I/II	PGA conjugated to the hydroxyl group of camptothecin via a glycine linker. Solubility of camptothecin is increased, preventing opening of the lactone ring. Drug loading around 33–35 wt%.	[166,171,172]
Delimotecan	CMD-T2513 conjugated	T-2513 (camptothecin analogue)	Solid tumours	Phase I	T-2513 bound to CMD through a Gly-Gly-Gly linker, with a molecular weight of 130 kDa. Drug loading between 3–6 wt%.	[135,173]
Taxoprexin	Polymer-drug conjugate	Paclitaxel	Various cancer	Phase II/III	2′-O-acyl conjugate of paclitaxel covalently bonded to the essential natural fatty acid DHA by an ester bond. Tumor AUCs for Taxoprexin are 61-fold higher at equitoxic doses and 8-fold higher at equimolar doses than paclitaxel. Other formulation properties not found.	[174,175]

**Table 8:** Polymer-protein conjugates approved for clinical use and currently tested in clinical trials. FDA = U.S. Food and Drug Administration; Mw = molecular weight; PEG = poly (ethylene glycol); mPEG = methoxy-poly (ethylene glycol); FDA = U.S. Food and Drug Administration; G-CSF = granulocyte-colony stimulating factor; EPR = enhanced permeability and retention effect; Mw = molecular weight; ADI = arginine deiminase.

Product Name	Formulation	Drug/Therapeutic Agent	Treatment	Status	Formulation Properties	Ref.
Oncaspar	mPEG-protein conjugate	L-asparginase	Acute lymphoblastic leukemia	Approved by US FDA (1994)	Around 69–82 molecules of mPEG covalently conjugated to L-asparaginase. Increased half-life, sustained activity of L-asparaginase, reduced number of injections.	[123]
Neulasta	PEG-protein conjugate	Filgastrim	Chemotherapy induced neutropenia	Approved by US FDA (2002)	20 kDa PEG molecule covalently conjugated to the α-amino group of the *N*-terminal methionine residue of Filgrastim, recombinant methionyl human G-CSF. Prolonged in vivo persistence.	[127,176]
SMANCS	Polymer-protein conjugate	Neocarzinostatin	Hepatocellular carcinoma	Approved in Japan (1994)	Passive-targeting formulation based on EPR effect. Neocarzinostatin conjugated to poly (styrene-comaleic acid) with the Mw of 16 kDa. In vivo t1/2 is 19 min.	[158,177]
Pegasys	PEG-protein nanoparticles	Interferon-α 2a	Various cancer	Phase I/II/III	Recombinant interferon α-2a (Mw > 19,000 Da) covalently conjugated to PEG chain (approximate Mw = 40,000 Da). Improved plasma half-life and uptake by liver, reduced dosing interval, but without sustained release pattern.	[103,178,179]
PegIntron	PEG-protein nanoparticles	Interferon-α 2b	Various cancer	Phase I/II/III	Recombinant Interferon-α 2b covalently conjugated to single straight-chain molecule of PEG with an average Mw of 12,000 Da. 10-fold increasing of plasma half-life from without compromising tertiary structure or spectrum of activity of IFN-α-2b.	[158,180,181,182]
ADI-PEG20	PEG-protein nanoparticles	ADI	Various cancer	Phase I/II	PEG (Mw of 20,000) conjugated to ADI by a succinimidyl succinate linker. Prolonged half-life with around 50% of the specific enzyme activity.	[183,184]

**Table 9:** Protein nanoparticles approved for clinical use and currently tested in clinical trials. DT = diphtheria toxin; FDA = U.S. Food and Drug Administration; Mw = molecular weight; IL2 = interleukin 2; HSA = human serum albumin; NAB = nanoparticle albumin-bound; mTOR = mammalian target of rapamycin.

Product Name	Formulation	Drug/Therapeutic Agent	Treatment	Status	Formulation Properties	Ref.
Ontak	Protein nanoparticles	DAB389, truncated DT	cutaneous T-cell lymphoma	Approved by US FDA (1999)	DAB389, a truncated DT (the first 388 amino acid residues), as the toxin part. IL2 as the targeting part, could bind to high-affinity IL2 receptor expressed on the malignant cells and regulatory T cells. Mw = 58 kDa.	[185,186]
Abraxane	Protein nanoparticles	Paclitaxel	Various cancer	Approved by US FDA (2005)	Formed by lyophilized HSA and paclitaxel, 130 nm in diameter. Increased solubility of drug but without sustained release pattern. Drug loading 6.6 wt%.	[52,103,187]
ABI-008	Protein nanoparticles	Docetaxel	Prostate cancer	Phase I/II	ABI-008, a solvent-free form of docetaxel, is based on NAB technology. Reduced side effects by eliminating polysorbate 80.	[188,189,190]
ABI-009	Protein nanoparticles	Rapamycin	Various cancer	Phase I/II	Based on NAB technology. Rapamycin is a protein kinase inhibitor. Size around 100 nm.	[191]
Rexin-G	Retroviral expression vectors	Phospholipid/microRNA-122	Solid tumour	Approved by Philippine FDA (2007)	A nonreplicative-targeted retroviral vector which has a cytocidal cyclin G1 construct. Size around 100 nm.	[192,193]
HAS-MTX	Protein nanoparticles	Methotrexate	Transitional cell carcinoma	Phase II	Methotrexate convently conjugated with HSA (Mw = 67 kD), in1:1 molar ratio. Increased tumour uptake and subsequently release of drug in a time-dependent manner with half-life about two weeks.	[194,195]

**Table 10:** Nanocrystals and other nanoparticles currently tested in clinical trials. SCF = supercritical fluid; 2ME2 = 2-methoxyestradiol; NCD = NanoCrystal^®^ colloidal dispersion; NHL = non-Hodgkin’s lymphoma; SPIONS = superparamagnetic iron oxide nanoparticle; HfO2 = hafnium oxide; SBRT = stereotactic body radiation therapy; TNF = tumor necrosis factor; PEG = poly (ethylene glycol).

Product Name	Formulation	Drug/Therapeutic Agent	Treatment	Status	Formulation Properties	Ref.
**Nanocrystal**						
Nanotax	Nanocrystal	Paclitaxel	Peritoneal neoplasms	Phase I	Aqueous, stable nanocrystal suspension of paclitaxel. Naked, rod-shaped particles with 600–700 nm in size, based on SCF technology. A depot system, intraperitoneal administration provides the stable reservoir of paclitaxel, extended drug release, increased tumour exposure with reduced toxicity.	[196,197]
Panzem NCD	Nanocrystal	2ME2	Various cancer	Phase II	2ME2 reconstituted as a NCD, improved PK properties and antitumour activity. Enhanced anticancer activity when plasma 2ME2 exposure is constant, for example, using implanted osmotic pump or multiple oral administrations every day.	[198,199]
Theralux	Nanocrystal	Thymectacin	NHL	Phase II	Formulated by a photosensitive drug and a device designed to eliminate cancer cells (used outside the body). Drug would undergo photodynamic activation when cancer cells are exposed to visible light using Theralux device, resulting in the death of the cancer cells, minimized side effects and toxicity.	[200,201]
**Other Nanoparticles**						
NanoTherm	Iron oxide nanoparticles	NA	Glioblastoma	Approved in EU (2010)	Aminosilane-coated SPIONS for local hyperthermia to treat tumours. After injecting into tumours, an alternating magnetic field is applied to selectively heat the particles, leading to tumour microenvironment to be heated locally to 40–45 °C, resulting in cell death. Size around 15 nm.	[93,202,203]
NBTXR3	HfO2 nanoparticles	SBRT	Various cancers	Phase II/III	50 nm nanoparticle composed of crystalline HfO2 functionalised by negatively charged phosphate coating. NBTXR3 improves the efficacy of radiotherapy.	[204]
CYT-6091	Colloidal gold nanoparticle	TNF	Advanced solid tumours	Phase I/II	Multivalent drug with 26 nm on size, designed to actively sequester TNF in solid tumours. TNF and thiol-derivatized PEG covalently linked to the surface of the colloidal gold nanoparticles without binding between PEG-THIOL and TNF.	[17,127,205]
AuroLase	Silica-gold nanoshells coated with PEG.	NA	Head and neck cancer, prostate neoplasms	Without FDA-defined phases (trials of devices or behavioral interventions)	Designed to thermally ablate solid tumours after stimulation with a near-infrared energy source. Silica core acts as a dielectric core, gold shell has thermal ablation capability after absorbing strongly near-infrared light, PEG layer provides stability.	[206,207]

#### 2.2.5. Other Smart Sustained Release Nanoparticles

There are other types of nanoparticles developed for cancer therapy not covered extensively in previous sections: these include mainly gold nanoparticles, carbon nanotubes, quantum dots and copper oxide nanoparticles [156,157,208,209]. Briefly, Pramanik et al. [157] reported on the synthesis of copper complex-tethered gold nanoparticles with biotin decoration, showing targeted delivery to tumours cells and controlled release showing targeted delivery to tumour cells and controlled release by glutathione as a trigger [157].

#### 2.2.6. Clinical Use of Nanoparticles as Cancer Therapeutics: A Perspective

After decades of research, there are nearly a hundred nanomedicines that have been tested in clinical trials as anti-cancer treatments, with only a small percentage of them being approved and marketed (Figure 3B). Within the number of research papers reporting synthetic polymeric NPs as cancer therapeutics, only 1% of these reports cover the testing of polymeric nanoparticles in clinical trials. Liposomes are better represented, clinical trials coverage accounts for approximately 4% of all publications [210]. However, although approved and used clinically, side effects and discomfort in patients are still reported. For example, Doxil^®^ can accumulate not only in the tumour tissue, but also in other tissues such as skin, with reported adverse effects on the palms and soles of the feet [211]. Abraxane has poor drug release to solid tumours, with poor therapeutic index; whereas Daunoxome and Eligard have reported low drug loading, with the latter showing initial burst release, further reducing the required performance [9]. In addition to these concerns, issues reported with clinically used liposomes also include; (1) poor reproducibility during manufacturing, (2) leakage of loaded drugs [212,213] and (3) fusion of liposomes during storage [214]. Among the other NPs, dendrimers are poorly translated to clinical use mainly as a result of the complexity of their synthesis and the associated manufacturing costs, as well as their low drug loading [215]. Micelles also have many issues yet to be addressed as drug delivery systems (e.g., off-target delivery, poor sensitivity to stimuli, limited understanding of micelles-biological membranes interaction), hence requiring further studies and improvements [216]. As previously discussed, the main limitation of polymeric NPs as therapeutics is the burst release of loaded drugs, coupled with the low drug loading [217]. Several studies that aim to solve these drawbacks, and these will be extensively discussed in Section 3. Metal NPs are promising candidates as delivery vehicles, with the main limitations of non-biodegradability and poor clearance. Surface modifications of gold NPs have been used to alter their biodistribution, toxicity or pharmacokinetics, however this still requires further investigations for their use in patients [218]. Interestingly, these nanoparticles (e.g., gold) can be responsive to near infrared-mediated release mechanisms of surface conjugated therapeutics, which can increase the efficiency of delivery to the tumour [219]. Carbon nanotubes are highly hydrophobic particles, with agglomeration observed when suspended in the aqueous phase. Cytotoxic properties [220] and the propensity to produce inflammatory reactions and lesions in vivo [221,222] have been also reported, therefore few are the studies focusing on their use.

## 3. Strategies for Achieving Sustained Drug Release from Polymeric (PLGA, PLA, PLGA/PLA) Nanoparticles

Polymeric nanoparticles are formulated using either natural or synthetic polymers, as summarized in Table 11. Between these, PLA, PLGA and their blends (PLA/PLGA) are the most commonly used synthetic polymers to make polymeric nanoparticles. These polymers have been used in biomedical applications for more than 30 years and are known to be biodegradable, biocompatible, and non-toxic [40]. Polymer properties such as polymer composition (*w*/*w* ratio), molecular weight (Mw) and crystallinity determine nanoparticles properties and the resultant release profile [223]. Drugs can be physically loaded in the polymeric matrix, as well as being chemically linked to polymers via hydrolyzable bonds; the latter method is used to increase and/or control the drug loading in the nanoparticles. The type of drug loading method is known to impact on release; among the other factors influencing the release mechanism are the physico-chemical properties of the loaded drug(s) and of the polymer(s) used to manufacture nanoparticles [224,225].

**Figure 4:**
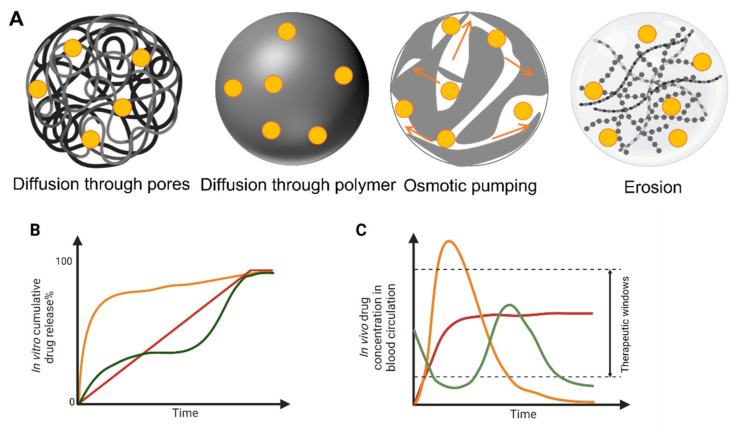
Drug release from polymeric systems. Mechanism of drug release through polymeric matrices (**A**). Drug cumulative release profiles in vitro of drugs from polymeric nanoparticles as drug delivery systems (adapted with permission from Ref. [217]. Copyright © 2016, American Chemical Society (**B**). Different cumulative release profiles represented as: monophasic pattern (red); bi-phasic pattern with burst release (orange); and tri-phasic pattern (green). Corresponding time-dependent concentration of drug measured in vivo for each release profile (**C**).

**Figure 5:**
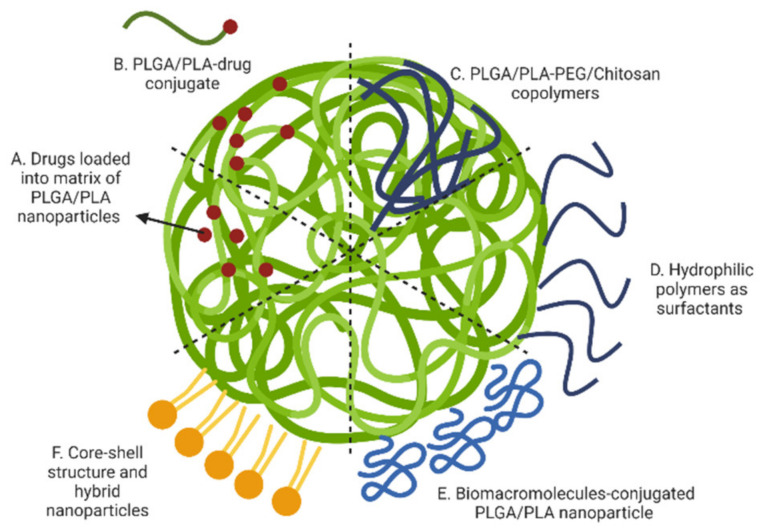
Polymeric nanoparticle modifications for prolonged drug release: (**A**) drugs loaded into matrix of PLGA/PLA nanoparticles; (**B**) nanoparticles made by PLGA/PLA-drug conjugate; (**C**) nanoparticles made by PLGA/PLA-PEG/Chitosan copolymer; (**D**) surfactants to cover the surface; (**E**) conjugation of biomolecules on the surface; and (**F**) core-shell structure and hybrid nanoparticles. (Created with BioRender.com, accessed on 23 September 2022).

Between the many PLA, PLGA and PLA/PLGA nanoparticles, four classical drug release mechanisms are reported in the literature: (1) diffusion through pores, (2) diffusion through the polymer matrix, (3) osmotic pumping and (4) erosion (Figure 4A). Diffusion through water-filled pores or channels, is possibly the first release mechanism to take place. Drug molecules diffuse through interconnected pores to the nanoparticles surface; such pores are more likely to be found in the nanoparticles formed using high Mw, hydrophobic, slowly swollen and degraded PLGA [228]. The second mechanism is the diffusion of the drug through the polymer matrix, described in systems when small hydrophobic drugs are loaded into the nanoparticles. In this case, the drug diffusion rate is dependent on the physical state of polymers: the rate is proportional to the glass transition temperature (Tg), and increases when the Mw of the polymer decreases [228]. The third release mechanism is driven by osmotic pressure, caused by water absorption within the nanoparticles (i.e., osmotic pumping). Degradation/erosion is frequently reported as a rate-controlled mechanism of release. Erosion is the principal drug release mechanism when low-Mw PLGA is used to form nanoparticles. In this case, bulk and surface erosion of PLGA nanoparticles promote drug availability, with further formation of pores to accelerate the release [228,229]. In water-based environments, typically multiple mechanisms for the release of loaded drugs may happen simultaneously, and the predominant mechanism could also change over time. All the above-mentioned mechanisms are valid when drugs are physically loaded into nanoparticles, and not representative of when drug-polymer conjugates are used to form nanoparticles [230]. Regardless of specific drug release mechanisms, there are typical drug release profiles reported for polymeric nanoparticles that are summarized in Figure 4B, typically comprising a fast initial burst release, followed by a slower second release phase [228,231]. The first fast release occurs when the nanoparticles are exposed to the dissolution medium, with the drug present on the surface of the nanoparticles being released according to its solubility. In this quick phase, no significant weight loss is reported as the polymeric matrix is not altered. In the second phase, hydrolysis of the polymer matrix occurs, and the remaining drug in the matrix is released following a single-phase zero-order drug release (Figure 4B, red line) [229]. The initial burst release is of utmost importance and needs to be carefully designed, as safety concerns must be considered if the released drug exceeds the toxicity threshold. Over the past few years, new manufacturing methods have been developed to prevent the initial burst release of the loaded drug, as this has been recognized as one of the main issues linked to polymeric nanoparticles [217]. A bi-phasic release profile, reported in Figure 4B,C (orange line), is observed when a first diffusion mechanism (driven by drug-polymer conjugate hydrolysis) is followed by degradation/erosion and hydrolysis release mechanisms [232]. A tri-phasic release profile can also be observed (Figure 4B,C, green line) in the case of heterogeneous degradation of polymeric nanoparticles [228]. Modifications designed into polymeric nanoparticles are summarized in Figure 5, these modifications aimed to enhance the control over drug release patterns and achieve a targeted and more sustained drug release profiles.

### 3.1. Drugs Loaded into Polymeric Matrix of Nanoparticles

The simplest strategy to load drugs into polymeric matrix (nanospheres) or nanoparticle cores (nanocapsules) is by physical loading during manufacturing. Several techniques can be used to manufacture polymeric nanoparticles, as described in Table 12.

Among the many studies reporting anti-cancer drugs successfully loaded in polymeric nanoparticles, some reported achieving prolonged drug release profiles over several weeks [240,241]. Mukherjee et al. [240] reported on PLGA nanoparticles prepared using a multiple-emulsion solvent evaporation method and loading tamoxifen citrate for anti-cancer treatment. The release of tamoxifen was studied over a few weeks at 37 °C in 1% hydroxypropyl beta cyclodextrin in phosphate-buffered saline pH 7.4, reporting 9.5 ± 0.1% tamoxifen released after 60 days as the slowest release profile between the formulations studied. Musumeci et al. [241] reported on the use of the solvent displacement method to load docetaxel into both PLA and PLGA nanoparticles; results showed a slow release of docetaxel in vitro (37 °C, phosphate buffer solution pH 7.4) from all the formulations, with 70–95% docetaxel released within 10 days. Guo et al. [242] reported on SN-38 loaded PLGA nanoparticles prepared using the oil-in-water solvent evaporation method. SN-38 release was tested at two different pH values, 1.5 and pH 7.4, it was reported a fast release of SN-38 at physiological pH.

When specific design considerations are taken into account, physical drug-loaded polymeric nanoparticles are reported to have high encapsulation efficiency (>80%) and avoid initial burst release [243,244]. However, these are case-specific formulations, and findings are difficult to extend to other similar systems without specific design. Overall, high drug loading and controlled release from physically loaded polymeric nanoparticles is an on-going issue for the majority of the formulations, with reported initial burst release up to 80–90% of the loaded drug happening within the first few hours. As previously mentioned, this phenomenon could cause severe systemic toxicities and coupled with low loading may prevent prolonged drug release at therapeutic levels [245].

### 3.2. Drug-Polymer Conjugated Nanoparticles

Drug-polymer conjugates are amongst the most explored options to solve drug burst release. The first example of drug-polymer conjugates for cancer treatment were proposed in 1975 by Ringdorf [246]. In this research, it was suggested to include a solubilizing domain in the conjugate spacer linking the polymer and insoluble (or poorly soluble) drugs to improve their bioavailability, as well as control and prolong their release in vivo [246]. The other main advantage of nanoparticles manufactured using drug-polymer conjugates is the potential to achieve increased drug loading.

Successful conjugation of doxorubicin to the terminal end group of PLGA via ester bond and further use of the conjugate to prepare nanoparticles was reported by Yoo et al., using the spontaneous emulsion solvent diffusion method [230]. Polymeric nanoparticles prepared using this doxorubicin-PLGA conjugate showed a greater drug loading (95.0 ± 7.5%) when compared physical loading of doxorubicin in PLGA nanoparticles (33.3 ± 4.3%). Moreover, doxorubicin-PLGA conjugate nanoparticles were able to release around 80% of loaded doxorubicin over 1 month (37 °C, phosphate buffered saline); while an initial burst release of doxorubicin (>50%) was observed within the first 24 h in physically loaded polymeric nanoparticles, in which all loaded doxorubicin was released after 5 days [230]. A different doxorubicin-PLA conjugate, which was reported by Tong and Cheng, formed doxorubicin-PLA conjugate nanoparticles were found to release 30% of loaded doxorubicin after 14 days (37 °C, phosphate buffered saline); whereas 90% of doxorubicin was released within 3 h in physically loaded polymeric nanoparticles [245]. As briefly shown, polymeric nanoparticles formulated using drug-polymer conjugates can offer the possibility to prolong/control the drug release profile by selecting an appropriate spacer/linkage, and have the potential to achieve a slow drug release over time, with an almost first-order release profile [247]. To add additional control over drug release kinetic, drug-polymer conjugates can be designed with chemical linkages to respond to pH and temperature variations [248].

### 3.3. Surface Modification Using Hydrophilic Polymers

The concept of nanoparticle surface modification was first conceived to solve interaction with body fluids, prolong plasma-retention time and achieve an extended-release profile [9]. Hydrophilic polymers are typically used to coat the surface of nanoparticles; these can be either absorbed to the surface (e.g., positively charged polymers like chitosan) or by using block or branched copolymers (e.g., PEG and its modifications) to formulate and manufacture nanoparticles [249].

#### 3.3.1. Use of Poly (Ethylene Glycol) (PEG)

PEG is a non-toxic, hydrophilic polymer commonly used to modify therapeutic molecules or delivery systems by covalent or non-covalent coupling [250]. PEG/PLA and PEG/PLGA copolymers are often used to fabricate nanoparticles [251]; PEGylation is another commonly used surface modification method for many types of nanoparticles, including polymeric nanoparticles [56]. PEGylation could; protect the nanoparticles from the reticuloendothelial system (RES) clearance in vivo, prevent nanoparticle aggregation in the bloodstream, and reduce the interaction between nanoparticles and plasma proteins and consequent degradation [252]. The presence of PEG was thought to decrease immunogenicity, improve the solubility, stability and prolong the retention half-life of the nanoparticles which benefits to reducing the dosing frequency whilst maintaining the required therapeutic index [253]. Recent studies, however, have shown the potential for in vivo production of anti-PEG-specific antibodies, which result in the rapid clearance of PEG from the body [254]. One of the first examples of PEGylated PLGA nanoparticles was reported by Gref et al. [255] showing that PEGylated nanoparticles had a significant increase of circulation time when compared to uncoated PLGA nanoparticles. Senthikumar et al. [256] compared in vivo performances of PEGylated and non-PEGylated PLGA nanoparticles and showed a longer half-life of PEGylated nanoparticles. In the case of the release of less toxic therapeutic agents, like curcumin, it was reported a similar prolonged release of both PEGylated and non-PEGylated nanoparticles, but PEGylated PLGA nanoparticles were found to have better distribution in vivo with promising pharmacokinetic parameters [257]. These results confirmed the role of PEG coating in steric protection with reduced uptake by mononuclear phagocyte system. Moreover, by controlling the physico-chemical properties of PEG/PLGA copolymers, Gu et al. [251] were able to fine-tune and control the drug release kinetics. By increasing the length of the PLGA segment, the rate of drug release in vitro could be prolonged to 3 days. However, the presence of PEG increases the interaction between nanoparticles with water, and as consequence, many studies showed faster in vitro drug release from PEGylated PLGA/PLA nanoparticles when compared to the nanoparticles without PEG [256,257,258]. The Mw of PEG is known to impact on drug release: for example, the inclusion of 5 kDa PEG resulted in a 0.6 µg mL^−1^ increase in maximum delivery of docetaxel when compared with nanoparticles coated with 2 kDa PEG [256]. On the contrary, the Mw of PEG does not impact nanoparticles circulation time, which is instead increased with the increase of the grafting density of PEGylated nanoparticles [252]. As the extracellular environment of the tumour is more acidic than normal tissues (i.e., pH < 7) [259], pH-sensitive nanoparticles can result in the shedding of the PEG coating and promote drug release at the tumour site. Therefore, PEGylation of nanoparticles can be an effective strategy for the fabrication of smart nanoparticle systems.

#### 3.3.2. Surface Absorption by Hydrophilic Cationic Polymers

Hydrophilic and cationic polymers, such as carbopol, dextran and chitosan [260,261,262], can be absorbed into the slightly negative surface of PLGA, PLA and PLGA/PLA nanoparticles to prolong drug release in physiological conditions. Chitosan is a natural and biocompatible polymer that has been used in many biomedical applications, including the fabrication and formulation of polymeric nanoparticles [263]. Chen et al. [264] studied the release of epirubicin from both PLGA nanoparticles and chitosan-coated PLGA nanoparticles, reporting that chitosan coating enabled a slower release of epirubicin in vitro during the first 24 h. Moreover, the presence of chitosan in chitosan-coated nanoparticles can promote and prolong adhesion of nanoparticles at the targeted site in vivo through ionic interaction with mucin, achieving a longer local drug release [262]. Another example of a natural polymer to coat PLA nanoparticles is dextran, which is responsible for reducing interaction with proteins, thanks to the presence of hydroxyl groups of dextran that increase the hydration layer around the nanoparticles, hence limiting protein adsorption. This hypothesis was verified by Verma et al., who demonstrated that pharmacokinetic parameters such as t1/2 of dextran-PLA nanoparticles were significantly improved in vivo when compared with PEGylated PLA nanoparticles [260].

### 3.4. Surface Modification Using Biomacromolecules

Surface modification of NPs with biomacromolecules, typically proteins, has been extensively studied, with wheat germ agglutinin and human serum albumin (HSA) the most commonly used in anti-cancer NPs, proving reduced initial burst release of loaded drugs [265,266]. An example is the use of HSA reported by Manoochehri et al. to increase circulation time and limit the burst release of HSA-conjugated PLGA nanoparticles loaded with docetaxel [266]. In this study, docetaxel loaded PLGA nanoparticles were found to have the typical release profile of physically loaded nanoaprticles, with initial burst release of docetaxel (40%, 10 h), followed by a sustained release over 12 days of the remaining drug in the PLGA nanoparticles. In the case of HSA-coated PLGA nanoparticles, a different docetaxel release profile was observed: a lower amount of drug was released over the first 24 h (20%), followed by a second slower release (75%, 8 days) and a final phase to release the remaining drug in nanoparticles (5%, 4 days) [266]. Another example of surface modification of PLGA nanoparticles was reported by Wang et al. [267] in which doxorubicin-PLGA conjugates were used to form nanoparticles via solvent-diffusion method, and further surface modification was achieved using PEG and Arg-Gly-Asp peptide sequence for active targeting of integrin expressing cancer cells. The study reports doxorubicin loading up to 85% in all formulations, with the limited initial burst release during the first several hours and prolonged doxorubicin release up to 12 days. Moreover, hybrid nanoparticles decorated with the selected peptide-sequence showed targeting ability of nanoparticles in tested cancer cell types of MDA-MB-231, B16F10 and MCF-7, when compared to uncoated nanoparticles.

### 3.5. Hybrid Nanoparticles and the Core-Shell Structure

Hybrid nanoparticles are defined as delivery systems composed of at least two types of biomaterials: a polymeric domain to form ‘the core’ and another component described as ‘the shell’ [268]. Materials selected to form ‘the shell’ have physico-chemical properties offering an extra layer to control drug delivery mechanism; being designed to enhance the therapeutic index at the site of interest. In the specific case of polymeric nanoparticles, materials selected as ‘shell’ components aim to prolong the release of the loaded-drug and often offer the possibility of controlling and/or triggering the release of the loaded drug (e.g., local delivery of chemotherapeutics in the tumour mass [269]). Polymer-lipid hybrid nanoparticles are one class of nanoparticles widely used for drug delivery applications. Polymer-lipid hybrid nanoparticles combine the advantages of both liposomes and polymeric nanoparticles, with reported higher drug encapsulation efficiency, sustained drug release profile, and the possibility to target specific diseased sites [270]. In these, the lipid ‘shell’ often acts as a diffusional barrier: slowing the release of the loaded drug and enabling delayed drug release once degradation of the polymeric core occurs. In the study reported by Wang et al. [271], the doxorubicin release profile was compared between doxorubicin-loaded lipid-PLGA hybrid nanoparticles and doxorubicin-loaded PLGA nanoparticles. The size of both nanoparticles was found to be similar, with the lipid shell nanoparticles slightly larger (364 ± 5 nm) than PLGA nanoparticles (342 ± 12 nm). The release of doxorubicin was slightly reduced by the presence of the lipid shell, with 70% of doxorubicin released from PLGA/lipid nanoparticles within a week compared to the 80% of doxorubicin released from PLGA nanoparticles. To enhance the functionality of nanoparticles, a multi-component shell can also be used to surround the polymeric core [272,273,274]. Cheng et al. [275] developed highly multifunctional PLGA nanoparticles loaded with paclitaxel for the treatment of lung cancer. In this study, the multicomponent shell is composed of a mix of gold nanorods, magnetic nanocrystals and quantum dots by conjugation for local and photothermally-triggered drug release. The study reports that the shell could reduce the amount of drug released from the hybrid nanoparticles when compared to PLGA nanoparticles. In another study, two coating layers were used for the co-delivery of doxorubicin and pEGFP (as a model DNA) using PLGA nanoparticles [271]. The PLGA hydrophobic core was firstly loaded with doxorubicin and then coated with an amphiphilic cationic PEGylated bilayer containing negatively charged nucleic acids. An additional folate-coated lipid layer containing cholesterol was added to stabilize the hybrid nanoparticles. Drug release was found to be slower in hybrid nanoparticles, with a prolonged release compared to drugs loaded in only PLGA nanoparticles. Moreover, the inclusion of folate on the surface allowed the targeting of tumour cells for the co-delivery of both chemo- and gene-therapies.

## 4. Physico-Chemical Properties and Formulation of PLGA/PLA Nanoparticles Impacting on Drug Release

### 4.1. Effect of Properties of Selected Polymer

#### 4.1.1. Mw of Polymer

The Mw of a polymer is defined as the sum of the atomic weights of individual atoms and indicates the average weight of the polymer chains [276]. As a general consideration, nanoparticles using polymers with different Mw are reported to have different degradation kinetics in physiological conditions, and typically the higher Mw the slower the degradation rate [217]. One of the first instances of research reporting the effect of PLGA Mw on the release of doxorubicin from nanoparticles, showed that nanoparticles fabricated with high Mw PLGA exhibited prolonged release compared to low Mw PLGA nanoparticles [230]. Similar results were found in a study comparing doxetaxel-loaded PLA nanoparticles and comparing release profiles when PLA with different Mw was used. In this study, PLA with higher Mw showed a higher burst effect while all of the drug release profiles showed a biphasic pattern [241].

#### 4.1.2. Composition, Crystallinity and Glass Transition Temperature (Tg) of Polymer

The presence of methyl side groups on PLA chains are responsible for its hydrophobicity, while PGA is more hydrophilic. Lactide rich PLGA copolymers are consequently less hydrophilic, absorb less water, and subsequently degrade more slowly [231]. The solubility of monomers could affect the degradation rate; hence the polymer composition contributes substantially to the solubility of the polymer. When in water, PLGA degrades by hydrolysis of its ester linkage. In a study by Wu and Wang, the effect of PLGA polymer composition on the resultant degradation rate was evaluated. A small library of polymers (PLGA with lactic acid (LA)/glycolic acid (GA) molar ratios set at 50/50, 65/35, 75/25, 85/15 and 100/0) was used in this study, and it was determined that the absolute value of the biodegradation rate constant increases with the increase of GA content [277].

Crystallinity and Tg of the polymer matrix, which depend on the polymer composition, have indirect effects on its degradation rate [278]. For the crystallinity, in classical stereochemical terms, the asymmetric-carbon within PLA has been typically classified as the D (PDLA) or L (PLLA) form. While, PLGA is generally an acronym for poly d, l-lactic-*co*-glycolic acid where d- and l-lactic acid forms are in fixed ratio [279]. And, because of the lack of methyl groups on the side chain, poly (glycolic acid) (PGA) is highly crystalline. When synthesizing the PLGA, the crystallinity and amorphousness depend on the ratio of monomers. However, there are studies showing conflicting results of the drug release influenced by crystallinity. Some groups showed the higher crystalline polymers could have higher mechanical strength and lower macromolecular chain mobility than the amorphous polymer which would have a slower degradation rate [279,280]. Conversely, there are also in vivo experiments that show higher crystallinity PLLA displaying an increased degradation rate. The reason for this discrepancy may be the high fabrication temperature (200 °C) during crystallization. It is known that PLLA is very sensitive to temperature; when the processing temperature is higher than the glass transition temperature, it will result in insubstantial degradation [278]. Also, different polymer compositions result in different Tg. In case of PLGA, the Tg increases with an increase in lactide content [217]. When the temperature is above the Tg, the polymer would be in a rubbery state, which contributes to higher mass transfer rates and diffusion of water/drug molecules throughout the polymeric matrix [281], results in faster release.

#### 4.1.3. Polymer End-Group Capping

Regardless of the Mw and composition (LA:GA ratio), each PLGA polymer contains carboxylic acid terminal groups, which can be end-capped as esters. Polymers with free carboxylic acid end groups hydrolyse and degrade faster when compared to the end-capped polymers, due to the hydrophilic characteristic of the carboxylic acid terminus [282]. Modifications of the PLGA end group (i.e., substitution of the ester with a carboxylic acid) increase the hydrophilicity of the polymer and absorption of water, hence higher in vitro degradation is observed. Of note, this modification could promote interaction between the carboxylic group and positively charged drugs, resulting in a slower release of the loaded drug compared to the capped PLGA [283].

### 4.2. Effect of the Drug

#### 4.2.1. Drug Characteristics

As described in the previous section, the characteristics of PLA, PLGA and PLA/PLGA blends used to formulate polymeric nanoparticles impact on the drug release profiles. Parallel to such considerations, the type of drug loaded into the system, its properties, and the interaction with the selected polymer, impact significantly on the drug release mechanism, and on the overall drug release profile. For example, the release of the loaded base or salt form of lidocaine in PLGA and PLA nanoparticles has been compared [284]. The study reports different release mechanisms, with accelerated hydrolysis of polyester links (i.e., via a base-catalysed reaction) when the base form of lidocaine was used. As a consequence, different drug release patterns are observed as a result of the combination of degradation rate and rate of water absorption within the polymeric matrix [284]. Similarly, when drugs with different physico-chemical properties (e.g., thiothixene, haloperidol, hydrochlorothiozide, corticosterone, ibuprofen, and acetyl salicylic acid) are loaded in PLGA nanoparticles, different drug loading and drug release profiles were observed, with the most hydrophilic drug contributing to the highest diffusion/swelling rate and degradation rate [285].

#### 4.2.2. Drug Loading (DL)

Drug loading (DL) is defined as the mass ratio of the drug and the drug-loaded nanoparticles and is typically expressed as a percentage [286]. Several manufacturing processes have been tested to increase the DL in polymeric nanoparticles; however, regardless the formulation and the manufacturing process used, it is difficult to achieve high DL values [217]. When drugs are physically loaded into nanoparticles, a greater burst release followed by a faster release rate is observed at high DL, with different values observed according to the properties of the loaded drug and of the polymeric nanoparticles [287,288]. Hydrophilic drugs follow the more general rule mentioned above, with a faster release and higher DL [289]. In the case of hydrophobic drugs, a higher DL was proven to slow the release of drugs, as the interaction with water is limited. For example, paclitaxel-loaded PLGA nanoparticles have shown faster release when the DL decreased [290].

### 4.3. Effect of Nanoparticle Properties

#### 4.3.1. Size of Nanoparticles

The size of nanoparticles was found to be critical for a range of NP properties including; degradation rate, drug release profiles, in vivo distribution and clearance, and cellular internalization. Therefore, formulation design and manufacturing processes are key steps to control in order to make nanoparticles with a known target size that are able to achieve the required therapeutic index at the tissue/site of interest [291]. Nanoparticles in the size range of 40–200 nm are reported to have prolonged circulation time, increased accumulation in tissues and decreased renal clearance [292], whereas nanoparticles larger than 200 nm have a higher rate of clearance [293] and nanoparticles smaller than 10 nm would be removed by renal filtration [294]. The release of drugs is highly dependent on the size of nanoparticles [291,295,296]. The high surface-to-volume ratio of small particles favours a higher degradation rate, and faster drug release [231]. Leroux et al. [297] studied the impact of size on drug release using savoxepine-loaded PLA nanoparticles: it was found that 50% of the savoxepine was released within 3 days in smaller particles (with the size of 300 nm), whereas the same amount of drug was released after 18 days in the larger ones (approx. 670 nm) with reduced burst release. Similar results were reported for coumarin-loaded PLGA nanoparticles, fabricated in three different sizes (200 nm, 500 nm, and 1000 nm), with the fastest release (5-times higher) found for smaller nanoparticles when compared to the larger nanoparticles [296]. Micro-particles, such as the FDA-approved medicine Lupron^®^ Depot (a PLGA-based technology), is a commercially available therapeutic, with micro-particles able to perform controlled release of leuprolide acetate between 1 to 6 months [103].

The size of nanoparticles also determines the site of accumulation and/or clearance when injected in vivo. Yadav et al. [298] compared the injection of two formulations of etoposide-loaded PLGA nanoparticles with sizes of 105 nm and 160 nm. Results showed that smaller nanoparticles (105 nm) were retained in the blood at higher concentrations, but accumulated more in the bone and brain tissues. Accumulation in tissues was found to be negligible when using larger nanoparticles.

#### 4.3.2. Shape of Nanoparticles

The shape of nanoparticles also impacts properties such as the degradation rate, drug release, targeting ability and cell internalization. For example, flat particles, such as rectangular disks or rods, present zero-order release, while more three-dimensional particles present a degradation profile that depends on how the shape (hence the surface) changes over time [299]. Ellipsoid particles were found to have reduced macrophage uptake, with the potential to have a prolonged circulation time and improved targetability [300]. In a study by Gratton et al., the internalization of different particle shapes in Hela cells was compared, reporting that particles with a size larger than 100 nm and with rods/ellipsoid shape have the highest uptake (with uptake rods/ellipsoid > spheres > cylinders > cubes) [301]. In the case of nanoparticles smaller than 100 nm, spherical particles were the most internalized particles. However, the interaction between non-spherical nanoparticles and cells may be more complex; and other geometrical parameters (e.g., short axis, long axis) may interact differently with cell surface receptors [302].

#### 4.3.3. Surface Charge of Nanoparticles

Surface modifications that impact the surface charge of NPs may also impact the drug release profiles. Yang et al. compared the release profiles of thienorphine-loaded PLGA nanoparticles as a function of surface charge (i.e., zeta potential, ζ). It is reported about 23.84 ± 1.43% thienorphine was released from pristine PLGA nanoparticles, and 14.29 ± 1.24% was released from the positive-charged nanoparticles [303]. The surface charge also as a pivotal role to direct nanoparticle interaction with the cell membrane, and subsequent uptake (e.g., endocytosis). Cationic nanoparticles (ζ > 0) tend to better adhere to the cell membrane, because of the presence of the anionic nature of phospholipids, proteins and glycans present on the plasma membranes of the target cells [304,305]. However, when comparing particles of similar composition and size, cationic nanoparticles were determined to have increased toxicity with respect to particles with a neutral surface charge [304].

#### 4.3.4. Fabrication Condition of Nanoparticles

Multitudes of protocols have been previously developed to fabricate polymeric nanoparticles (as described in Section 3.1). Fabrication design and methods, impact on drug release, size, surface charge, drug loading, and all together finally control the efficacy of the nanoparticles in vivo. PLGA nanoparticles manufactured by emulsification and nanoprecipitation have a direct correlation between size and organic solvent fraction used during manufacturing [306]. Huang and Zhang [307] evaluated different organic solvents (i.e., acetonitrile, acetone and tetrahydrofuran) and their impact on the size of PLGA nanoparticles prepared by nanoprecipitation, finding increased size with tetrahydrofuran > acetone > acetonitrile. As the diffusion coefficient of the three solvents is in the opposite order, it is assumed that the diffusion coefficient of the solvent in water could be used to predict the size of obtained nanoparticles. A similar study evaluated the influence of temperature on particle size (as the diffusion coefficient varies with temperature), reporting approximately a 10 nm decrease in particle size with an increase in temperature [307].

## 5. External Stimuli for Triggered-Drug Release in Polymeric Nanoparticles

**Figure 6:**
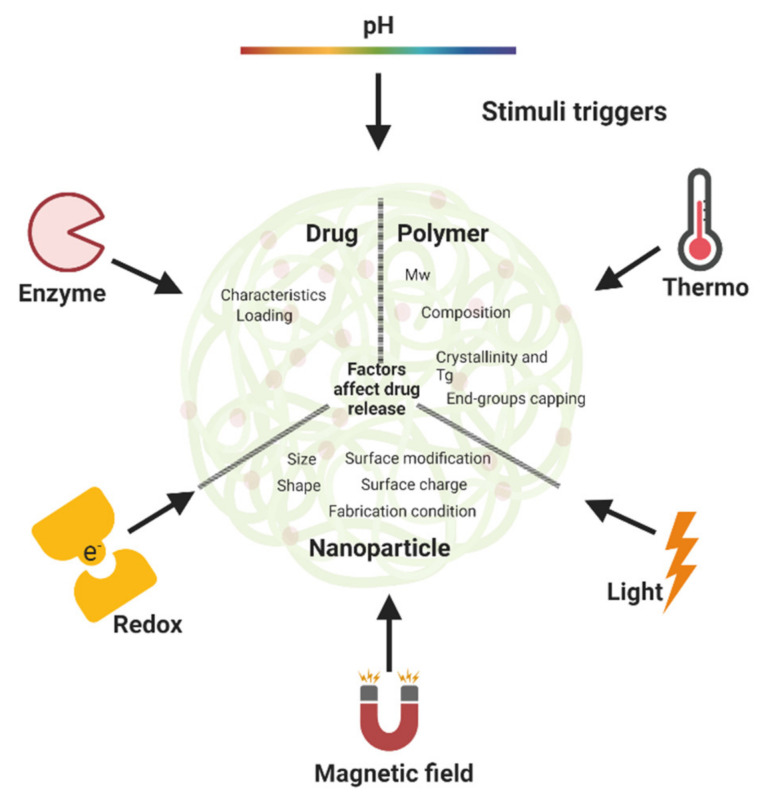
Internal factors and external stimuli that affect drug release from smart PLGA/PLA nanoparticles. (Created with BioRender.com, accessed on 23 September 2022).

Smart sustained release nanoparticles are purposely designed to respond to specific stimuli. With specific reference to cancer treatment, smart NPs enable activation and/or release of therapeutic agents at the tumour site in response to tumour microenvironment variables (e.g., pH, enzyme concentration, temperature) [308,309] or to external stimuli (e.g., temperature, ultrasound, magnetic fields) [310,311] (Figure 6).

### 5.1. pH-Triggered Release

One of the most reported methods to enable the release of therapeutics in the tumour microenvironment is by using pH-responsive nanoparticles. pH-responsive nanoparticles are designed to release the loaded therapeutic at specific pH values typical of the tumour microenvironment (pH < 7). For example, Zhao et al. prepared multifunctional doxorubicin-loaded PLGA nanocapsules by using an oil and water (*o*/*w*) emulsion method. Release of doxorubicin is achieved via electrostatic interaction, due to the weaker interactions between doxorubicin and PLGA at lower pH values [259]. Another example is a pH-responsive nanoparticle technology designed by Clawson et al., in which the PEG-coated lipid-polymer hybrid nanoparticles contain an ester bond linkage that is pH-sensitive [312].

### 5.2. Thermo-Triggered Release

Variation of temperature is also used to trigger the release of therapeutics from nanoparticles in both in vitro and in vivo settings. ThermoDox^®^, a PEGylated liposome formulated using a thermosensitive lipid (with Tg in the range of 40–45 °C), is currently under Phase III for breast cancer treatment [27,28]. Using a similar approach, PLGA nanoparticles with a magnetite core and thermo-responsible shell were reported by Wadajkar et al. for the controlled release of curcumin and doxorubicin when temperature is increased above 41 °C, with proposed use in hyperthermia treatment [313]. Biodegradable thermo-sensitive polymers (i.e., PLGA-PEG-PLGA triblock copolymer) were also proposed as carriers because of the reported safety in vivo [314].

### 5.3. Light-Triggered Release

Many technologies are reported to release payloads from nanoparticles upon controlled exposure to an external light source, with release controlled by light wavelength and intensity as an “on-off” switch [14,315]. Yang and colleagues reported a light-triggered system formulated with doxorubicin-loaded PLGA nanoparticles coated with a gold layer, which was responsive to light irradiation in the near infrared region (i.e., 820 nm). Upon irradiation, doxorubicin is quickly released due to the resultant heat generation (i.e., above Tg of PLGA) [316]. In a similar study, Part et al. [317] also showed the doxorubicin release could be triggered by near-infrared irradiation from gold layer-modified PLGA nanoparticles and, compared to the non-modified PLGA nanoparticles, modified nanoparticles showed a slower release without irradiation. Another strategy to fabricate light-responsive polymeric nanoparticles is the inclusion of light-responsive groups in the polymer used. PLGA can be conjugated to pendant nucleophiles protected by the o-nitrobenzyl, which can be cleaved using 1 mW/cm^2^ UV light. Upon exposure, the modified polymer becomes more hydrophilic, allowing water to penetrate the polymeric matrix and further promoting the hydrolysis of the polymer backbone itself, as well as releasing the loaded therapeutics [318]. Furthermore, another strategy to achieve light-triggered release is by using a photosensitive drug. For instance, verteporfin (a photosensitive drug) loaded with PLGA nanoparticles showed better tumour responses with early exposure time to red light in mice tumour models [319]. However, the use of light-sensitive nanoparticles is limited as they cannot be used for non-invasive applications due to the limited penetration of light in human tissues, therefore invasive procedures (e.g., surgery) are required in the case of treatment of deep tissues (e.g., abdomen) [320].

### 5.4. Magnetic Field-Triggered Release

Exposure of nanoparticles to external magnetic fields can be used to (1) induce an increase of temperature in the nanoparticles, which in turn triggers the release of the drug, and (2) attract nanoparticles to a specific volume in the target tissue, hence increased the dose of released therapeutics and improved efficacy of the treatment at the site of interest [321]. Although magnetic-responsive nanoparticles are a promising technology, a challenge remains to adjust the intensity of the external magnetic field and direct this within a specific volume of the body and with sufficient penetration, allowing nanoparticles to accumulate in the tumour tissue and release therapeutics in highly controlled events [315]. So far, iron oxide nanoparticles are one of the most studied magnetic nanoparticles [322]. Iron oxide can be loaded into polymeric nanoparticles, with particles successfully proven to respond to magnetic fields. For example, PLGA nanoparticles co-encapsulating magnetic iron oxide nanoparticles and doxorubicin, produced using a single emulsion evaporation method, were described by Guo et al. In this study, a core-shell structure with magnetic iron oxide core and PLGA shell was shown to release doxorubicin upon exposure to an external magnetic field [269].

### 5.5. Redox-Triggered Release

Glutathione is a low Mw thiol (consisting of three amino acids) that is abundant in mammalian cells and has highly effective antioxidant activity. The R-SH structure is able to reduce di-sulphide bonds and could be used as a strategy to promote the release of chemotherapeutics in tumour cells (in which the concentration of glutathione is at least 4 times higher than that of normal cells) via a redox-responsive mode [14]. One example using this strategy are nanoparticles of PLGA-PEG co-polymers linked by dithiylethanoate esters. Such nanoparticles not only showed a faster uptake in lung cancer in vitro models, they also allowed the sustained release of loaded therapeutics when compared to the control group (i.e., nanoparticles made of PLGA-PEG di-block copolymer without disulfide bonds). When exposed to glutathione, a fast and triggered release of loaded therapeutics was observed for redox-responsive nanoparticles, indicating PEG cleavage, PLGA degradation and disassembly of nanoparticles due to the presence of glutathione [323]. Other co-polymers designed with a similar strategy were described in work by Shen et al., in which hyaluronic acid-modified disulfide was crosslinked with PLGA-polyethyleneimine. In the study, siRNA and paclitaxel were loaded into the nanoparticles and released at high concentrations of glutathione [324].

### 5.6. Enzyme-Triggered Release

Enzymes are proteins involved in many biological processes, with the main action in regulating cell functions and maintaining homeostasis. During cancer formation, homeostasis may be disrupted, and consequently, enzyme profiles vary between normal and tumour tissues. Higher concentrations of specific enzymes present in the tumour microenvironment can be used to selectively trigger the release of therapeutics from nanoparticles. Examples of enzymes, dysregulated in tumour microenvironment and used for release of drugs in enzyme-responsive nanomedicines for cancer treatment are: metalloproteinases (MMPs), gelatinases, hyaluronidase-I, esterases and phospholipase A2 [321,325]. Typically, enzyme-responsive nanoparticles are modified with peptide sequences, cleaved by specific enzymes (e.g., MMPs). Dorresteijn et al. synthesized polymeric nanoparticles using PLA-peptide-PLA triblock copolymer, and showed controlled drug release in vitro in presence of MMP2 [326]. Mi et al., formulated coumarin-6 loaded PLGA-PEG nanoparticles coated with MMP2 peptide substrate, showing that 40% of coumarin-6 was released over 6 h in the absence of MMP2, whereas 80% of coumarin was released in the presence of MMP2 [327]. Although successful, this approach has some drawbacks, for example, the potential release of drugs in an off-target site where an enzyme is present at a high concentration, without specifically targeting the tumour mass and/or cancer cells [327]. Another concern is the heterogeneous expression of enzymes in different cancer types, and at different stages of progression, making it challenging to extend the use of a treatment to a large cohort of patients [18].

## 6. Challenges of Sustained Release from Smart Nanoparticles

As discussed in this review paper, drug loading and release of therapeutics with required doses at the site of interest (e.g., tumours) are common problems across all different types of nanoparticles. In the past few years, several refinements and improvements have been implemented on the formulation of nanoparticles for tumour treatment and led to a precise control over drug loading, tissue-targeting and prolonged release. However, achieving the required therapeutic concentrations of specific therapeutic agents specifically in tumours is still challenging when nanoparticles are dosed in patients. The design of responsive nanoparticles to external stimuli (e.g., “on-off” switchable properties) has proven advantageous not only from a manufacturing perspective, but also for faster in vitro to in vivo translation and improved therapeutic efficacy. Among the many challenges to hasten the bench-to-bedside translation of smart sustained release nanoparticles, the gap between discovery and consistent results in vivo is the principal limiting factor. Formulation design, manufacturing and further scale-up remain challenging for many technologies. Optimized processes are used to manufacture small batches, however variability in nanoparticles obtained may affect their properties, hence their efficacy. Besides repeatability issues and batch-to-batch variation, the manufacturing methods currently used for the preparation of nanoparticles require revisiting, with regard to the design quality of products produced. This could impact on the selection of materials used in the formulations, and the processes themselves. For example, during the preparation of polymeric nanoparticles, organic solvents are typically removed by dialysis and/or evaporation methods, which are inefficient and time-consuming [328]. The lack of standardization in many processes for the manufacturing of nanoparticles still causes high variability in their size, drug loading, etc. [328]. The increase of functionalities in smart sustained release nanoparticles requires additional manufacturing steps: that should allow not only a higher control over drug loading, necessary to achieve the required therapeutic windows, but also over nanoparticle’s physico-chemical and surface properties, as the composition, size, geometry, and surface chemistry of nanoparticles is known to impact on the interaction between nanomaterials and biological systems [329]. Also, the selection of in vitro and in vivo models should be thoroughly considered to minimize variation and maximize the validation of nanoparticle performance. Many studies testing the efficacy of nanomedicine in biological systems focus only on specific cases, limiting further use of nanoparticles [330]. The lack of understanding of such interactions still limits our knowledge of efficacy and safety of smart sustained release nanoparticles both in vitro and in vivo. Although nanoparticles could improve the delivery of therapeutics, there are several complex biological barriers to overcome. The mononuclear phagocytic cell responses, the route of administration, cancer types and high intra-tumour pressure are examples known to limit the targeting, accumulation and efficacy in vivo [320]. Personalized nanomedicines have been considered promising candidates, as they have the potential to bypass limiting factors for efficacy, such as age, genetics, cancer type and stage of cancer development. Exhaustive studies of the additional genetic and epigenetic biomarkers could assist in the development of new types of targeting components. Triggered release of selected therapeutics by external stimuli may further improve efficacy. However, research in this field requires careful monitoring, as the types of energy used may damage surrounding healthy tissues [9].

So far, pH- and thermo-responsive nanoparticles have limited responsiveness windows, as there can be limited variation between normal and tumour tissues, with intrinsic local temperature and pH variation dependant on the tumour microenvironment and patient physiology. Therefore, it is challenging to accurately design responsive nanoparticles that could translate to clinical settings, as further personalized pathophysiological research of patients is required [9]. Additionally, it may be also important to re-consider the route of administration of nanoparticles. Traditionally, nanoparticles have been formulated for oral [331], nasal [332] or intravenous delivery [333]; innovative products using other delivery routes (e.g., dermal and mucosal application, pulmonary drug delivery) have greater potential to solve off-target accumulation and delivery, with higher accumulation to the tumour sites, and hence improve the treatment efficiency and reduce the damage to the surrounding healthy tissues. Finally, and of utmost importance, it is necessary to implement a regulatory framework specific to nanomedicines. In 2014, the US FDA released guidance about the application of nanotechnology. So far this is the only official regulation that can be referenced [320], which is far from sufficient, especially considering the interest they have attracted as potential clinical therapeutics in recent years [334,335,336]. The application of nanotechnology differs from those of conventionally-manufactured products, therefore regulatory counterparts in different countries need to share perspectives and information on the regulation of nanotechnology products.

## 7. The Future of Sustained Release Smart Nanoparticles: Microfluidics?

In conclusion, polymeric and liposomal nanoparticles have much potential as methods of targeted cancer treatment. However, due to the difficulties outlined above, translation is often a timely and complex process due to the discrepancy between laboratory and clinical production [337]. As previously mentioned, batch-to-batch variability and the scale-up of nanoparticle production have presented significant challenges in the development of marketable and clinically translatable nanotechnologies. Microfluidics, a recently popular method for the fabrication of nanoparticles, uses chips containing a variety of conformations of micro-capillary tubes to direct the flow of aqueous (drug containing) and organic phases (lipid or polymer containing) downstream, and eventually their mixing. This method uses the principle of cross-flow chemistry, whereby organic and aqueous phases undergo a forced interaction by directing continuous laminar flow through chips containing capillary tubes [338,339]. Different chips contain differing junctions and can be used to achieve increased control over nanoparticle dimensions and characteristics, by controlling mixing times and flow rates of the two phases (organic/aqueous). This method enables precise control over manufacturing parameters and enables the upscale production of non-variable nanoparticle batches [340]. Gkionis et al. (2020) used microfluidics to produce DMPC/DSPC (with cholesterol and DSPE-PEG2000) liposomes and stated that using this method, these formulations can be used to produce batches containing up to 20 mL of liposomal suspension [339]. Many accounts of microfluidics-assisted fabrication of nanoparticles exist within the literature, with some authors reporting superior encapsulation efficiencies of nanoparticles formed using microfluidics, when compared with those produced using other methods [339,341,342,343]. This demonstrates the suitability of using microfluidics as an emerging and viable alternative to more traditional manufacturing processes. Movement towards this production method has the potential to yield large amounts of nanoparticles with improved payload uptake. Microfluidics has demonstrated marked potential for future widespread use with its rapid and controlled method of nanoparticle fabrication. As we continue to explore effective nanoparticle fabrication methodologies, microfluidics appears a strong contender for the production of clinically translatable nanotechnologies.

## Figures and Tables

**Table 11:** The definition, features and examples of natural polymers and synthetic polymers used for the manufacturing of drug delivery systems [40,226,227]. PCL = Poly(ε-caprolactone); HPMA = *N*-(2-hydroxypropyl)-methacrylamide; PLA = poly (lactic acid); PLGA = poly(lactic-co-glycolic acid).

Polymer Type	Definition	Features	Commonly Used Polymers
Natural polymers	Raw materials that naturally occur in the biological environment	Biocompatible, Biodegradable, Low immunogenic levels, Low-cost, Allowing chemical modifications	Gelatin, dextran, chitosan, collagen, albumin, heparin
Synthetic polymers	Macromolecules synthesized using different primary materials (e.g., natural products, oil).	Highly predictable physical properties such as solubility, permeability and rates of biodegradation. Increased the pharmacokinetics and circulation times of incorporated therapeutic substances.	PCL, HPMA, PLA, PLGA

**Table 12:** Different techniques used for preparation of drug loaded polymeric nanoparticles [231,233,234,235,236,237,238,239]. *w*/*o* = water in oil; *w*1/*o*/*w*2 = water in oil in water; EE% = encapsulation efficiency; *o*/*w* = oil in water; *o*/*o* = oil in oil; SESD = spontaneous emulsion solvent diffusion.

Techniques	Preparation Methods	Type of Drug	Particular Features/Advantages	Disadvantages
*w*/*o* phase separation	Emulsify aqueous drug phase with polymer-dissolved organic phase	Hydrophilic	High encapsulation efficiencies for hydrophilic drugs, due to their insolubility in organic solvents.	Need to handle and dispose of oil; difficult to control condensation.
*w*1/*o*/*w*2 emulsion	Emulsify aqueous drug phase (*w*1) with polymer-dissolved water-immiscible phase (*o*). Mix *w*1/*o* with aqueous phase (*w*2).	Hydrophilic	Manufactured using high- or low-energy technologies; easily control the size distribution.	Poor EE% for small molecular weight (escape to the *w*2 phase) during the encapsulation process.
*o*/*w* solvent evaporation	Emulsify polymer-dissolved organic phase droplets into aqueous phase. Remove solvent by evaporation, emulsion droplets solidified into nanoparticles.	Hydrophobic	Easily adapted for hydrophobic drugs; good reproducibility; ease of scaling up the manufacturing process.	Use of volatile halogenated organic solvents, toxic solvents used; solvent residual.
*o*/*w* solvent extraction	Add excess of quench solvent such as water to the *o*/*w* emulsion to promote quenching organic solvent into the aqueous phase	Hydrophobic	Use of non-halogenated solvents.	High volumes of waste stream produced; difficult to remove solvent completely; nanoparticle aggregation.
*o*/*o* emulsion	Emulsify drug and polymer-dissolved organic phase (*o*1) with a continuous oil phase (*o*2)	Hydrophobic	Preparation method for water-insoluble drug, using non-halogenated solvent.	Issues to dispose and/or recycle of waste oil during manufacturing.
*o*/*w* salting-out	Emulsify drug and polymer-dissolved organic phase with an electrolyte-saturated aqueous phase	Hydrophobic	Using non-halogenated solvent; low energy mixing device	Using large quantities of salting-out agents which need to be recycled such as salts/electrolytes.
Nanoprecipitation (solvent dialysis method)	Mix polymer-dissolved organic phase with an aqueous phase through a low energy mixing device.	Hydrophobic	Use of non-halogenated solvent; one-step method for loading water-insoluble drugs; low-energy mixing device.	Low concentration of polymer in the dispersed phase; nanoparticles may aggregate because of unremoved solvent.
SESD	Dissolve polymers in mixture of water miscible and water immiscible solvent; nanoparticles are formed by emulsification and solvent evaporation	Hydrophobic	Use of pharmaceutically acceptable organic solvents with no need of high-pressure homogenizers.	The binary solution contains a halogenated solvent.
Spray drying	Spray solid-in-oil dispersion or water-in-oil emulsion in a stream of heated air	Hydrophilic	Fast and easy method with a small number of parameters; suitable for industrial fabrication	Nanoparticles may adhere and/or agglomerate to the inner walls of the spray dryer.
Microfluidics	Mix two phases of liquids in a microfluidic device with the microchannel at least one dimension smaller than 1000 μm	Hydrophilic/Hydrophobic	Control formation processes precisely; desired EE%; multiple drugs loading capacity; low energy mixing device	Limitation for scaling-up the process.

## Data Availability

Not applicable.
